# ‘We All Work Together to Vaccinate the Child’: A Formative Evaluation of a Community-Engagement Strategy Aimed at Closing the Immunization Gap in North-West Ethiopia

**DOI:** 10.3390/ijerph15040667

**Published:** 2018-04-03

**Authors:** Tracey Chantler, Emilie Karafillakis, Samuel Wodajo, Shiferaw Dechasa Demissie, Bersabeh Sile, Siraj Mohammed, Comfort Olorunsaiye, Justine Landegger, Heidi J. Larson

**Affiliations:** 1 London School of Hygiene & Tropical Medicine, Keppel Street, London WC1E 7HT, UK; emilie.karafillakis@lshtm.ac.uk (E.K.); Bersabeh.sile@gmail.com (B.S.); heidi.larson@lshtm.ac.uk (H.J.L.); 2 Assosa Referral Hospital, Benishangul Gumuz Regional State, Assosa, Ethiopia; wodajo.samuel@yahoo.com; 3 International Rescue Committee, Bole Sub-City, 813 Addis Ababa, Ethiopia; Shiferaw.Demissie@rescue.org (S.D.D.); siraj.mohammed@rescue.org (S.M.); 4 International Rescue Committee, New York, NY 10018, USA; comfort.olorunsaiye@rescue.org (C.O.); justine.landegger@rescue.org (J.L.)

**Keywords:** immunization, vaccination, community engagement, increasing uptake, Ethiopia, child health, qualitative research

## Abstract

The role of community engagement (CE) in improving demand for immunization merits investigation. The International Rescue Committee developed a CE strategy to implement a vaccine defaulter-tracing tool and a color-coded health calendar aimed at increasing uptake of immunization services in north-west Ethiopia *(*‘The Fifth Child Project’). We report findings from a formative evaluation of this project. In May/June 2016 we conducted 18 participant observations of project activities, 46 semi-structured interviews and 6 focus groups with caregivers, health workers, community members/leaders. Audio-recordings and fieldnotes were transcribed, anonymized, translated and analyzed thematically using inductive and deductive coding. Additional data was collected in November 2016 to verify findings. The project was suitably integrated within the health extension program and established a practical system for defaulter-tracing. The calendar facilitated personalized interactions between health workers and caregivers and was a catalyst for health discussions within homes. At the community level, a regulation exercise of sanctions was observed, which served as a deterrent against vaccine default. Pre-existing community accountability mechanisms supported the CE, although varying levels of engagement between leaders and health workers were observed. The benefits of shared responsibility for immunization were evident; however, more transparency was required about community self-regulatory measures to ensure health-related discussions remain positive.

## 1. Introduction

Despite significant progress in increasing the number of infants immunized worldwide, global immunization coverage has remained steady at 84–86% since 2010 [1]. Over 35% of World Health Organization (WHO) member countries, including Ethiopia, are struggling to meet the 90% coverage target for the third dose of diphtheria, tetanus and pertussis-containing vaccine [1]. To break through this stagnation, increased attention is being paid to the role of community-engagement (CE) approaches, whereby health systems engage or partner with community beneficiaries to address supply and demand factors [2]. 

It is well known that contextual factors and the degree to which community members understand and trust vaccination can affect vaccination behaviors [3,4,5]. The potential role of CE approaches in improving demand for immunization services is less well researched. Initial assessments of community-based interventions are positive and further evaluations planned [6,7,8,9]. A recent review indicates that interventions designed and co-managed with community members are more likely to be successful [2]. A relevant example from Pakistan is a project that involved community members in evidence-based discussions about immunization, which resulted in stimulating local action to address barriers and increase vaccine uptake [10]. Cooperation between administrative and political leaders at district and community level has also been identified as a key driver for immunization performance, as have community health workers promoting and supporting vaccination activities where they live, and tailoring immunization activities to community needs [11]. 

This paper contributes to this emerging evidence by reporting qualitative findings from a formative evaluation of a CE strategy and related tools (the ‘Fifth Child Project’) developed by the International Rescue Committee (IRC). The defined aims of the Fifth Child Project (FCP) were to close the immunization gap and improve the uptake of maternal and child health services in north-west Ethiopia. 

A separate report [12] elaborates on contextual factors (e.g., geography and health system), describes the implementation, theory of change (ToC) and intended outcomes of the FCP, and includes a quantitative analysis of routine immunization and project data. 

### The Project and Its Context

IRC is a humanitarian non-governmental organization that has been supporting the regional health infrastructure in Benishangul-Gumuz Regional State (BGRS) in north-west Ethiopia since 2003. Access to health care services in this developing regional state is variable and coverage for the third dose of pentavalent vaccine (diphtheria, tetanus, pertussis, Haemophilus influenza type B, hepatitis B) was only 41.7% in 2011 [13]. To help close the immunization gap and improve the uptake of maternal and child health services, IRC developed and implemented the FCP in 114 *kebeles* (smallest administrative unit in Ethiopia) in Assosa and Bambasi woredas (districts) in BGRS between 2014–2017. The FCP and related tools were designed in consultation with the Regional Health Bureau, Woreda Health Officers (WoHO), and community leaders. IRC continuously monitored the progress of the FCP and iteratively refined it until 2017. 

The FCP was integrated within the health extension program (HEP) launched in BGRS by the Ethiopian Ministry of Health in 2009. HEP’s mandate is to deliver 16 essential health packages in a community-centered manner. It consists of a network of health posts located in *kebeles* where preventative and primary clinical services are provided. Two government-salaried female health extension workers (HEWs) (health extension workers are a core component of the health extension program that was introduced in Ethiopia in 2003. They are young women selected by their communities to be trained to provide primary health services at health posts) run the health posts, conduct household visits and organize immunization outreach services. Members of the Health Development Army (HDA) (the Health Development Army was introduced in 2011 as an initiative for social change. It consists of female volunteers supervised by health extension workers. Each volunteer (a Health Development Army member) must run a model household and is responsible for advising 5 neighboring households, and they are supported by leader (Health Development Army Leader) who is responsible for a group of 30 volunteers) support the HEWs by reinforcing positive health practices at household level (Box 1).

Box 1 **The Fifth Child Project: tools and community-engagement approach.**

The Fifth Child Project consisted of two tools and a CE strategy. One tool was a vaccine defaulter-tracing tool (DTT) and the other a color-coded health calendar, called *Enat Mastawesha* (Amharic for “mother’s reminder”). The DTT was a simple carbon-copy registration form used at the health post to record, by village, basic infant/caregiver information and vaccines missed. Its use helped HEWs and Health Development Army leaders (HDALs) identify homes that HDAs should visit/follow-up. Pregnant women and women with infants received the *Enat Mastawesha* from HEWs and HDAs. The calendar included colored stickers which HEWs attached to a mother’s perinatal and an infant’s immunization appointment dates. It also portrayed key health messages in pictorial format, e.g., when to seek medical assistance urgently during pregnancy. The CE strategy consisted of training HEWs and HDAs in using the tools, interpersonal communication skills, and promoting shared responsibility for immunization at household and *kebele* command post level (*Kebeles* are led by an executive body of five to seven cabinet members including chairman (elected), three council members, a *kebele* manager, development agent, health extension officer and school director. The manager is reasonably educated, salaried and appointed by the *woreda*. *Kebele* command post meetings occur weekly or bi-monthly and as part of their agenda they review and evaluate the performance of different sectors (e.g., health, education) and subsequently the *kebele* managers submit reports to the relevant *woreda* offices.), with the aim of leveraging improved follow-up of unimmunized infants and timely uptake of scheduled vaccines. To ensure that immunization services were available and timely, additional systems support (e.g., cold chain maintenance, transport and training) was also provided. IRC hypothesized that the use of the DTT and calendar within a larger CE strategy—that directly involves caregivers and specifically implicates existing community leadership structures—would address identified barriers to vaccine uptake.

## 2. Materials and Methods

### 2.1. Study Design 

The purpose of the qualitative study was to evaluate the FCP in terms of integration into the local health system, community co-management and the acceptability and utilisation of the FCP tools. A series of interviews, focus group discussions (FGDs) and observations were conducted between May–July 2016. As part of this research, IRC developed a ToC (Supplementary Materials), which depicted the inputs and activities required and key assumptions made for the FCP to achieve specified outputs and outcomes. The ToC provided a framework for evaluating the project and findings were used to refine the ToC and improve the implementation of the FCP.

### 2.2. Setting

Three *kebeles* from BGRS were selected for the evaluation to include study sites with different population size, cultural and religious affiliation and distance from an urban centre (Table 1). Mugfude and Jematsa were mainly inhabited by indigenous Muslim populations and Amba 17 by Christian settler groups. The primary income-generating activity was agriculture, supplemented by gold mining in Mugfude and Jematsa. 

The sample frame within these sites included people who played a role in the FCP: caregivers, HDA(L)s, HEWs, HEW supervisors, nurses (seconded to health posts), *kebele* leaders (KLs), other community leaders (e.g., teachers) and WoHO. We included all HEWs/nurses and all KLs from the *kebeles* and key WoHOs responsible for immunization in these *kebeles* and used purposive sampling to select a set number of HDA(L)s, caregivers and other community leaders with maximum variation in terms of age, education level, gender and caregiver relationship to the infant across the three *kebeles*. While this mix of recruitment strategies was the only option due to the limited number of HEWs, nurses and KLs available in the three *kebeles*, the possible impact on the representativeness of the different sampling groups was taken into account by analysing and reporting data by type of participant.

### 2.3. Data Collection

A local team of research assistants (‘research team’) from BGRS with English, Amharic and Rutana language skills were trained to conduct interviews, FGDs and observations in order to limit the influence of the European London School of Hygiene and Tropical Medicine (LSHTM) researchers on participants. The local research team lead (SW) was well known from the community as he had previously worked as a clinical officer in the regional hospital’s maternity wing. This facilitated rapport with the participants, including caregivers and district-level leaders, but could have also influenced some of the results. TC was present for a few initial interviews and focus groups to provide support and feedback to the newly trained research team, which could have influenced some of the participants’ responses.

Interviews and FGDs took place in private settings and lasted 1–1.5 h. The interview style was semi-structured to cover pre-defined topics and shape exchanges according to interviewees’ roles, experiences and responses. FGDs were conducted with caregivers (mothers and fathers) and HDAs to facilitate discussions about immunization, the tools, and their engagement among themselves. With the interviewees’ permission, the research team collected basic socio-demographic information, compiled field notes, and audio-recorded interviews and FGDs. 

Participant observations, conducted to obtain more information about how the DTT and calendars are used along with the community’s engagement, focussed on two FCP activities: (1) monthly command post meetings with *kebele* leaders, HEWs, HDALs, and representatives of youth and women groups; and (2) HEW/HDA routine visits to caregivers’ homes. The research assistants conducted the observations in pairs and compiled field notes summarising the event, the type and number of people present, the purpose of the activity, and discussions held. The LSHTM researchers were not present during the observations to limit their influence on interactions and activities, but this also created a limitation as the local research team, including SW, was not fully trained on this methodology that would have required a different set of skills and more time and immersion in the field. 

### 2.4. Analyses

The research team transcribed the audio recordings into Amharic with secretarial support. The transcripts were anonymised by allocating a numerical identification to each participant and storing these separately from the participant database. The transcripts were translated from Amharic to English by a firm in Addis Ababa, emailed securely to TC and EK and uploaded to NVivo, a qualitative data analysis software program. The approach to data analysis was thematic [14] and used the FCP’s ToC as a reference point. TC and EK developed a coding framework by drawing parent codes from the topic guides deductively and developing sub-codes inductively. They coded the first five transcripts separately, and met to compare findings and to start developing the framework. A report on data collection and findings compiled by SW, and field notes from the observations, were also used to inform the interpretation of the data and the identification of themes. TC coded the remaining transcripts, meeting several additional times with EK to refine the framework. SW also commented on the framework in bi-monthly phone calls. Codes were organized together with quotes from the transcripts to compare and contrast the data, including the recurrence of certain themes and the terminology used by participants. Categories and typologies were drawn from the codes and developed into emerging themes to further discuss the meaning of the data. An Ethiopian researcher based in the UK (BS) supported this data analysis by conducting translation checks and acting as a cultural interlocutor (although she was not originally from BGRS, she worked closely with the local research team lead (SW)). 

This analysis and initial findings were discussed with members of the research team, IRC FCP implementers, district and regional health offices, health extension workers and health development army members and some research participants during dissemination activities in BGRS in November 2016. During this time, SW, BS and TC conducted additional interviews with a WoHO, two KLs, two HEWs and two FGDs with caregivers (all mothers) and HDAs to verify some analyses, specifically those relating to the use of the *Enat Mastawesha* and maternal literacy levels and the application of community-agreed sanctions for non-immunization. This data was analyzed as described above. 

### 2.5. Ethical Considerations

Potential participants received a study information letter in Amharic from the local research team and had the opportunity to ask questions before agreeing to be interviewed. Verbal consent is the preferred practice for obtaining agreement for participating in research in this region, hence participants audio-recorded a statement citing their name, date and willingness to participate. To preserve participants’ confidentiality, all transcripts and observation sheets were anonymised, and only the research team had access to the names of participants. The LSHTM Observational and Interventions Research Ethics Committee (Ref 10542) and the Regional Health Bureau of BGRS in Ethiopia approved this research (Ref 674/□□ m-o1).

## 3. Results

### 3.1. Study Participants 

A total of 46 interviews, six FGDs (3 with HDAs and 3 with caregivers, including two with mothers and one with fathers to obtain additional information from men in households), 15 observations of routine visits by HEWs and HDAs to caregivers (five per *kebele*), and three command post meetings observations (one per *kebele*) were conducted from May–July 2016 (see Table 2). In addition, five interviews and two FGDs were conducted as part of a data-verification exercise in November 2016. The purpose of this exercise was to address questions that had arisen during the analysis and to collect additional data to verify the analytical thinking and interpretation of the data. 

### 3.2. Analytical Themes

Table 3 presents a summary of the key themes identified from the interviews, FGDs and observations, a short definition, and selected sub-themes. These themes are further described and explored in the sub-sections below.

#### 3.2.1. Community Acceptance of the Fifth Child Project

*Kebele* leaders and HEWs reported that some community members were initially suspicious of the FCP thinking that, “the government was forcing them to do something that was not in their interest.” (KL, #45). These concerns were addressed in orientation meetings with *kebele* leaders, which were crucial for community acceptance of the FCP.

“Had the community and the leaders not accepted Enat Mastawesha as a positive thing, it would not have been admitted into our houses. In the same way, a door is opened with keys, the calendar was introduced with the consensus of the community.”

(Female caregivers, FGD)

It was evident from interviews with caregivers, KLs and health workers that initial hesitations abated as the benefits of the FCP became more apparent. HEWs reported that the project tools decreased their workload and enabled them to reach families in remote areas. Mothers reported that the calendar helped them maintain better control over their child’s health, and KLs observed a change in attitudes towards immunization.

#### 3.2.2. *Enat Mastawesha*: Catalyst for Health Dialogue within Homes

The calendar was described by all types of interviewees as an excellent personalized reminder for mothers that had reduced health workers’ workload, increased demand for immunization, and facilitated timely uptake of vaccines. It was attached to walls in caregivers’ homes and served as a communication aide for health workers and a catalyst for health-related discussions between family members.

“Previously it was considered taboo for women to discuss obstetric and gynaecological issues with her husband. But since the training with the Enat Mastawesha we have learnt that everything can be discussed openly within the marriage, with health workers and even with friends. Due to these open discussions, women have been able to receive help as soon as their symptoms arise.”

(Male caregiver, SSI, #7)

Most caregivers stated that mothers and fathers were involved in vaccine decision-making and, where possible, HEWs would explain the calendar to both. Caregivers liked the fact that the calendar included pictures and found the stickers useful, although a few reported that their primary school-aged children had removed the stickers to play with them. Older children helped their mothers with less education to read the calendar as did some of their husbands. Illiterate users could identify calendar dates but were unable to read the titles of the health education pictures. Despite this, they were mainly able to relay and act on the key messages conveyed by the pictures. 

#### 3.2.3. Shared Community Responsibility for Immunization

HEWs stated that the defaulter tracing system and the related DTT had improved their access to vaccination data and enabled them to count and identify defaulters in a more systematic manner. Similarly, WoHOs valued the system as an information source that facilitated collaboration with KLs. HDALs coordinated the follow up of unimmunized infants, enlisting the support of *kebele* and village leaders where necessary. The level of involvement of community leaders ranged from strategic assistance, e.g., sending local militia to find infants displaced due to parents’ gold-mining activities, to active follow-up and in some instances enforcement. 

Caregivers generally supported KLs’ involvement in defaulter tracing, stating it promoted compliance since leaders “were heard by the community”. However, they also highlighted their own role in promoting vaccination uptake as part of the 1-to-5 HDA model household health network.

“The community brings children to vaccination centres by themselves. Except (for) my husband, no one orders me to take my child to get vaccinated.”

(Female caregiver, SSI, #19)

Pre-existing accountability mechanisms facilitated the community co-management of the FCP. HEWs and HDAs were answerable to the *kebele* command post and were required to jointly address service gaps. Varying levels of collaboration with KLs were reported, and key factors that influenced engagement were: (1) the frequency of command post meetings; (2) whether HEWs tabled activity reports; and (3) KLs’ priorities and commitments. KLs’ specific input included reviewing vaccination planning, highlighting service gaps, community mobilisation, and supporting defaulter tracing. Related to defaulter tracing, we found evidence of community-agreed sanctions (e.g., monetary fines, cautions by local cabinet) in two *kebeles*. “If a parent lets his child miss a vaccination or a husband hinders or does not help his wife give birth at a health facility, he can be detained for 24 h. Such penalties are agreed by the consensus of the community. (…) The community thinks to pay a penalty of up to 500 birr, for failing to attend for health services is a good thing. This shows their eagerness for the service, however, so far nobody has been penalised.” (Male caregivers, FGD).

In the data verification exercise, KLs and HEWs confirmed that sanctions were part of a community self-regulation strategy agreed by *kebele* members and applied by the *kebele* cabinet without input from WoHOs or the IRC (sanctions were not included in the FCP CE strategy). They were issued where there was evidence of complete disregard of guidance provided by HEWs and HDAs and were not limited to vaccine default but also covered health facility non-attendance for childbirth. The latter was the only instance cited in which a penalty had been issued and collected. In general, the threat of sanctions served as a deterrent, a last resort for persistent non-immunisers.

“The penalty is only there to stop mothers wasting the HEWs’ time and as far as I know this penalty has never been issued in this kebele for vaccine defaulters. I am only aware of one lady who had to pay because she gave birth at home, putting the child’s health at risk.”

(KL, Follow-up SSI, #9)

#### 3.2.4. Demand for and Access to Immunization

Many interviewees reported a shift in public opinion about immunization over the past 5–8 years—from reluctance to high levels of vaccine acceptance. This was linked to the introduction of the HEP and resulted in greater demand for vaccines.

“Families feel like these vaccinations are their human right, and if vaccinations don’t take place as scheduled, they come to us to ask when the vaccines will be given.”

(WoHO, SSI, #28)

In gauging the specific role played by the FCP in this change of attitude, health workers highlighted the personalized interactions it facilitated. These interactions gave caregivers the opportunity to express any concerns they had about vaccination. Logistical support also meant that HEWs could run vaccine outreach events in remote villages and make connections with families that were hard to reach. Interviewees were keen for the FCP to expand but also stressed the need to address remaining infrastructural barriers, e.g., staff shortages, lack of fridges, vaccine stock-outs. 

#### 3.2.5. Health System Integration 

WoHOs reported that the FCP became a valuable and integral part of the existing HEP. KLs and other health workers agreed that the project had created systematic ways of encouraging the uptake of immunization in their communities.

“This project has introduced a new way of working. (…) Having the Enat Mastawesha and the follow up form is useful for reminding women of their check-up and vaccination dates. This all in one format has proved to be very useful.”

(WoHO, SSI, #14)

The FCP was well adapted to the health infrastructure; HDAs represented a trusted conduit for communication and follow-up at household level; and HEWs were well positioned to explain, administer vaccinations, collaborate with KLs, and coordinate vaccine-defaulter tracing. 

## 4. Discussion

These findings demonstrate the benefits, challenges and nuances involved in promoting shared community responsibility for immunization. The FCP facilitated discussion and vaccine-related activity within households, at *kebele* health and command posts, and at district level. The follow-up of unimmunized children became more personalized and the ‘*Mama Matawesha*’ was a catalyst for health discussions between mothers and fathers within homes as well as between health workers and families. It promoted joint parental responsibility for vaccine decision-making and health workers were able to address parents’ questions more specifically within the home environment. Leveraging the involvement of fathers in vaccine decision-making has been shown to increase the likelihood of children completing the vaccine schedule, hence this is a distinct benefit of the FCP [15]. The emphasis placed on supporting intrapersonal communication in the FCP also corresponds with findings from recent research in Liberia that found that community members preferred this to social mobilization activities (e.g., mass communication via radio, press, parades or flyers) [6].

The FCP promoted collaboration between the HDA, HEW, KLs and WoHOs and established a systematic way of following up unimmunized children. Such interactions between community leaders and health officials are key to improving immunization performance [11]. They are also a means of identifying infrastructural weaknesses (e.g., lack of fridges to maintain a cold chain) that can deter vaccine supply. Once identified, gaps in provision need to be addressed, since inaction can reduce the usefulness of community co-managed interventions [2]. Although this is a formative evaluation and results cannot be attributed to the intervention, an analysis of routine immunization data shows that between January 2013 and December 2016, pentavalent-3 coverage increased from 63–84% in Assosa, and from 78–93% in Bambasi. The increase in vaccination coverage in both woredas was statistically significant [12].

The FCP was well adapted to the HEP infrastructure and pre-existing *kebele* accountability mechanisms supported FCP-related CE, although varying levels of engagement across *kebeles* was noted. An unexpected by-product of increased activity within the HEP were *kebele*-level internal discussions about community-agreed sanctions for non-immunization. This practice can be viewed as a form of community self-regulation possibly linked to how *kebeles* (as observed by IRC authors) seek to outperform each other to obtain recognition for achieving HEP targets (e.g., *kebeles* where most households have latrines receive MoH sanitation milestone-achieved billboards). This self-regulation provided evidence that the FCP had fostered shared responsibility, but it also raised questions: (i) who is qualified to determine the type of sanctions that should be applied; (ii) if monetary, who collects fines and how should they be invested; and (iii) at what level of the health system should these types of measures be ratified?

The concept of community is used and defined slightly differently depending on geographic, cultural and disciplinary contexts (e.g., development, bio-medical research, public health promotion) and there is no universally accepted definition [16]. With reference to CE in biomedical research, Tindana et al. [17] present different models with varying gradients of participation from simple information-sharing to more active community input and control. Popay [18] with reference to health improvement in the UK uses the following definition: “Community engagement refers to the process of getting communities involved in decisions that affect them. This includes the planning, development and management of services, as well as activities, which aim to improve health and reduce health inequalities”. With regard to immunization programmes, Sabarwal et al. [2] stress the importance of the health system working directly with service beneficiaries (e.g., community members) to address supply and demand side factors; it is not enough for an intervention to be community-based, it must also actively involve community members. In order for CE to be useful and transformative there needs to be transparency about how responsibility and power will be shared [19]. Our findings support this and denote the importance of delineating who is involved in decision-making, resource allocation, and instigating change. 

Core to this is two-way communication. This may explain the positive findings from Anderson et al.’s research in Pakistan that assessed whether evidence-based discussions with community members increased vaccine uptake [10]. Community members were given the opportunity to discuss the findings of a baseline survey on the costs and benefits of childhood immunization, and were asked to draw up local action plans to address challenges and barriers. These resultant plans included promoting discussion about vaccination within households, sharing transport to vaccination points, and providing care for some children while parents took others to be vaccinated. 

The FCP succeeded at promoting discussion about immunization within households and at a community level; however, proposed community self-regulatory measures were unexpected. Whilst proposed sanctions indicated that *kebeles* had embraced responsibility for immunization, they also raised ethical questions about coercion and enforcement of vaccination, even if the rationale of the proposed sanctions were to ensure that valuable health resources were afforded due respect. Patryn and Zagaya [20] discuss these questions in a review of sanctions (welfare cuts, fines, exclusion from schools and theme parks and restrictions on freedom) applied in different countries. They suggest an alternative approach, whereby individuals are required to contribute to treatment costs if they contract the illness for which they refused immunization. This argument corresponds with the desire to protect and respect health resources observed in this study, and provides an alternative approach to sanctions, which may not be effective and are hard to apply fairly [21]. 

Finally, we acknowledge that these findings are not generalizable; however, they may be transferrable to similar settings and have wider public health implications. The involvement of local researchers in data collection and transcription minimised response bias and ensured that verbal data was timely and accurately documented. Additional translation checks by BS during the data analysis supported analytical rigour. 

## 5. Conclusions

This study indicated that the Fifth Child Project intervention, implemented in north-west Ethiopia with the aim of improving vaccination coverage, was well integrated into the local health system and contributed to improved awareness and demand for vaccination by facilitating the follow-up of unimmunized children. The *Enat Mastawesha* calendar enabled health discussions between family members, health workers and community leaders. The calendar also allowed more personalized and focused discussions between health workers and families and played a role in achieving timely vaccination. The intervention was successful at promoting parental responsibility for vaccination but may have contributed to *kebeles* creating and agreeing on their own sanctions against non-vaccinated individuals. These findings point to the need for more transparency around community self-regulatory measures in future immunization programs. Involving communities and relevant leaders in immunization programs can be very effective, however the lines of responsibility and the authority to determine and execute different measures needs to be clarified to ensure that such measures are in line with national health policy and do not deter underserved families from vaccinating their children. 

## Figures and Tables

**Table 1:** Composition of selected study sites.

*Kebele* (Woreda)	Distance from Woreda Main Town	Health Extension Workers (HEWs)/Health Post	Health Development Army/Leaders (HDAs/HDALs)	Total Population	Infants <1 Year
Mugfude (Assosa)	58 km	2/1	42/14	2011	62
Amba 17 (Assosa)	32 km	2/1	18/15	1035	32
Jematsa (Bambasi)	24 km	2/1	43/11	1432	46
Total		6/3	103/40	4478	120

HEWs: Health Extension Workers; HDAs: Health Development Army member; HDALs: Health Development Army Leader.

**Table 2:** Number of interviews and focus groups by area and participant type (excluding verification interviews and focus group discussions (FGDs)).

Area and Participant Type	Type of Interview	
	Semi-Structured Interview (F = Female, M = Male)	Focus Group Discussion
*Kebele:* Amba 17		
Caregiver	4 (2F, 2M)	1 (*n* = 10F)
Nurse	1 (M)	
Health Extension Worker	2 (F)	
Health Extension Worker Supervisor	1 (M)	
Health Development Army Leader (1 in 30)	1 (F)	
Health Development Army Member (1 in 5)	2 (F)	1 (*n* = 8F)
Teacher	1 (F)	
*Kebele* Leaders	2 (1M, 1F)	
*Kebele: Jamatsa*		
Caregiver	4 (2F, 2M)	1 (*n* = 8F)
Nurse	1 (M)	
Health Extension Worker	2 (F)	
Health Extension Worker Supervisor	1 (M)	
Health Development Army Leader (1 in 30)	1 (F)	
Health Development Army Member (1 in 5)	2 (F)	1 (*n* = 8F)
Teacher	2 (M)	
*Kebele* Leaders	1 (M)	
*Kebele: Mugfude*		
Caregivers	5 (3F, 2M)	1 (*n* = 8M)
Nurse	1 (F)	
Health Extension Worker	2 (F)	
Health Extension Worker Supervisor	0	
Health Development Army Leader (1 in 30)	1 (F)	
Health Development Army Member (1 in 5)	2 (F)	1 (*n* = 7F)
Teacher	1 (M)	
*Kebele* Leaders	2 (1F, 1M)	
*Woreda* level interviewees		
*Woreda* Health Officers	2 (M)	
Expanded Program of Immunization Officers	2 (M)	
Totals	46	6

**Table 3:** Key themes identified from the data.

Theme	Data Captured under This Theme	Sub-Themes
**Community acceptance of the Fifth Child Project (FCP**)	Acceptability of the FCP (defaulter-tracing tool (DTT), *Enat Mastawesha* and the community engagement strategy) from its initiation and over the course of time, by community-level stakeholders. Reasons for acceptance, hesitation, and reluctance to be involved.	Initial hesitations about the FCP Community leaders as gatekeepers ○ Value of orientation sessions Perceived barriers of the FCP Perceived benefits of the FCP over time Views on continuation and expansion of the FCP FCP alignment with existing health extension program (HEP)
***Enat Mastawesha*: catalyst for health dialogue within homes**	Practical usability of the calendar, its role as a health communication tool within homes (with attention to the Health Development Army structure of 1 model and 5 neighbouring households) and between health extension workers and infant caregivers.	Usability of the calendar in practical terms ○ Perspectives of health workers/caregivers Literacy, comprehension and the use of the calendar Vaccine and health-decisions making ○ Catalyst for health dialogue
**Shared community responsibility for immunization**	Evidence of the involvement of community leaders in FCP activities and the level of responsibility they were assigned and assumed.	Facilitators for collaboration with community leaders ○ e.g., pre-existing administrative health accountability mechanisms (including responsibility for public health) Barriers to collaboration with community leaders ○ e.g., priority setting and irregular meetings Community leader’s involvement in defaulter tracing ○ Perspectives of caregivers/leaders/health workers Consequences and stigma related to vaccine default
**Demand for and access to immunization**	Data that indicates/discusses the contribution of the FCP to increasing demand for and access to vaccination.	Changes in demand due to health system strengthening in last decade FCP role in strengthening health care infrastructures FCP role in facilitating outreaches in hard to reach areas FCP role in facilitating personalized interactions between health workers and caregivers
**Health system integration**	Data that provides insights into how the FCP was aligned with the Ethiopian primary care system and related activities.	Creation of systematic ways of increasing vaccine uptake HEP program staff and Health Development Army (HDA) trusted conduits for delivering FCP activities

## Supplementary Materials

The following are available online at http://www.mdpi.com/1660-4601/15/4/667/s1, Supplementary Materials 1: Study Protocol with the Fifth Child Project’s Theory of Change; Supplementary Materials 2: Data collection tools; Supplementary Materials 3: Picture of the defaulter tracing tool; Supplementary Materials 4: Picture of the *Enat Mastawesha* calendar.
