# Supplementary material for: ‘We All Work Together to Vaccinate the Child’: A Formative Evaluation of a Community-Engagement Strategy Aimed at Closing the Immunization Gap in North-West Ethiopia
Source: Int J Environ Res Public Health. 2018 Apr 3;15(4):667. doi: 10.3390/ijerph15040667 (PMC5923709; doi:10.3390/ijerph15040667)
Supplement: Supplementary file 1 [file ijerph-15-00667-s001.zip › Supplementary materials_Chantler et al_protocol and data collection tools_260218.pdf]

A formative evaluation of *'The Fifth Child Project'*: A new initiative to close the childhood immunisation gap in Benishangul-Gumuz Regional State in Ethiopia

Principal investigators
Shiferaw Demissie[1], Tracey Chantler[3], Anne Langston[2], Heidi Larson[3]

Co-investigators
Azeb Abtew[1], Dereje Assefa[1], Emilie Karafillakis[3], Lilian Kiapi[2], Justine Landegger[2], Siraj Mohammed[1], Isaac Mwase[1]

1. International Rescue Committee (IRC) Ethiopia
2. International Rescue Committee (IRC) Headquarters New York
3. London School of Hygiene & Tropical Medicine

Funder: 3ie International Initiative for Impact Evaluation

Protocol History

AMENDMENT NO.	PROTOCOL VERSION NO.	DATE ISSUED	AUTHOR(S) OF CHANGES	DETAILS OF CHANGES MADE
Original version	1	30/12/15		
1	2	29/02/16	Tracey Chantler & Emilie Karafillakis	1. TC added as a PI (page 1)<br>2. Minor changes to number of interviews conducted (page 15)<br>3. Minor changes to participant observation section (page 17)<br>4. Minor addition to verbal consent process (page 16, 20, and 29)

Table of Contents

Investigators roles and responsibilities 3
Study Background 4
Introduction 4
Study Context 5
Geographic and socio-demographic profile 5
Figure 1: Benishangul-Gumuz Regional State in Ethiopia 5
Factors influencing vaccine uptake in the study area 6
Health system considerations 6
IRC's Community Engagement Strategy: 'The Fifth Child Project' 7
Cross-cutting community engagement approach 7
Project components 8
1. Communication & behaviour change 8
2. Tracing and follow-up of defaulting and unimmunized children 9
Rationale for the formative evaluation 10
Purpose and design of the formative evaluation 11
Study aim 11
Study objectives 11
More detailed evaluation questions 12
Study Design 12
Evaluation team 13
Sampling Frame 13
Methods 14
System for monitoring immunisation and project data 14
Qualitative interviews and focus groups 14
Participant recruitment 16
Participant sampling 16
Participant observation of project activities 17
Data management and analysis 19
Ethical considerations 20
Dissemination and reporting of results 21
Timeline 22
References 23
Appendix 1 24
Project implementation feasibility and the underlying theory of change 24
Appendix 2 27
The Colour Coded Health Calendar – 'Mama Matawesha' 27
Appendix 3 29
Study Information Letter 29

Investigators roles and responsibilities

IRC Headquarters

Implementation

Justine Landegger:	Technical monitoring and field support
Lilian Kiapi:	Technical monitoring and field support

Justine Landegger and Lilian Kiapi will provide technical monitoring and field support in the areas of project implementation, analysis of routine data and support for evaluation data collection and stakeholder engagement

Evaluation

Anne Langston:	Technical support for the evaluation

IRC Ethiopia

Implementation

Siraj Mohammed:	Manager of the project, based in BGRS

Siraj Mohammed is based in Assosa, and will be responsible for coordination and liaising with the Regional Health Bureau, Zonal Health Department, Woreda Health Offices, primary health care units, and health posts in the targeted areas. He will be responsible for periodic monitoring field visits to ensure quality of data collection.

Evaluation and implementation

Shiferaw Demissie:	Technical oversight and responsible for the evaluation on the ground

Shiferaw Demissie will be responsible for the overall technical oversight and management of the IRC-lead components of this formative evaluation, integrity of project design, and monitoring to ensure quality and coordination.

Azeb Abtew:	Technical assistance and evaluation
Dereje Assefa & Isaac Mwase:	Technical assistance

Azeb Abtew and Dereje Assefa are based in Addis Ababa and will be responsible for the planning, implementation, and monitoring of the project. They will also be responsible overseeing the recruitment and supervision of field staff who will collect and translate the evaluation data. Isaac Mwase is IRC Ethiopia's learning and accountability coordinator based in Addis, he will provide support on monitoring aspects of the project activities and help with the formative evaluation.

LSHTM

Heidi Larson & Tracey Chantler:	Evaluation oversight
Tracey Chantler & Emilie Karafillakis:	Data analysis and evaluation

Tracey Chantler and Emilie Karafillakis will be responsible for overseeing the evaluation activities with the support of IRC Ethiopia colleagues. They will train the external field

workers who will collect the data and will lead the analysis of the routine and qualitative data. Heidi Larson will review and support their work.

Study Background

Introduction

Ethiopia's national coverage of the third dose of the pentavalent combined diphtheria, pertussis, tetanus, hepatitis B, and Haemophilus influenza type B vaccine (Penta3) at 12-23 months of age is 37% and the dropout rate between the first and third doses of this vaccine is 43%(Central Statistical Agency [Ethiopia] and ICF International, 2012). Ethiopia is one of the 35/194 WHO Member States which have not succeeded in reaching the Global Vaccine Action Plan's intermediate goal of reaching 90% national coverage with three doses of diphtheria-tetanus-pertussis containing vaccines by 2015 (WHO, 2015). Less than half of WHO African region states were able to meet this goal, and although some East African countries, like Eritrea, have surpassed the 90% threshold, rates in Ethiopia, Kenya, Uganda and South Sudan have stagnated and remained low (WHO, 2015).

In the search for novel ways to address low and stagnating vaccination rates and improve access to and utilisation of immunisation services increased attention is being paid to the role of communities, and community engagement (CE) strategies (Sabarwal et al., 2015). It is argued that communities should not be viewed passive recipients of immunisation services but actively involved in shaping vaccination programmes. The Global Vaccine Action Plan places significant emphasis on shared individual, community and governmental responsibility for immunisation against vaccine preventable diseases, and cites the following as one of its strategic objectives: "*Ensuring that individuals and communities understand the value of vaccines and demand immunization as both their right and responsibility*". (WHO, 2013, p.30)

It is well established that contextual factors and the degree to which community members understand and trust the immunisation process affect vaccination coverage rates (Rainey et al., 2011, Favin et al., 2012, Streefland et al., 1999). Less well documented is the effectiveness of CE strategies in addressing vaccine supply and demand factors and improving vaccine coverage. Evidence suggests that vaccine interventions that are designed and co-managed with community members are more likely to be successful, however more attention needs to be paid to evaluating and developing current CE practice (Sabarwal et al., 2015). Existing evidence has tended to focus on immunisation activities that aim to raise awareness and promote behaviour change by running education programmes targeted at infant caregivers or community health workers, or using a variety of communication tools such as improved vaccination cards or community-based posters (Jain et al., 2015, Owais et al., 2011, Ryman et al., 2011, Usman et al., 2011). Less attention has been paid to integrated CE strategies that seek to involve community members and leaders in identifying new ways of reducing the childhood immunisation gap and strengthening existing immunisation programmes. In their work on identifying key drivers for immunisation performance in Africa, LaFond et al stress the importance of co-operation between the health system and administrative and political leaders at district and community. They also highlight the value community-centred health workers who can promote and support vaccination activities in the places where they live, and emphasise the need to tailor immunisation activities to community needs.

This study aims to contribute to the emerging evidence base by conducting a formative evaluation of an integrated CE strategy that is being implemented by the International Rescue Committee (IRC) in two districts (Assosa and Bambasi) in Benishangul-Gumuz Regional State in north-west Ethiopia.

Study Context

Geographic and socio-demographic profile

Ethiopia has an estimated population size of 96 million (USAID, 2015) of which 82% live in rural areas where they rely on farming and livestock production (Central Statistical Agency [Ethiopia] and ICF International, 2012). It is one of the least developed countries in the world and is currently ranked 173/182 on the Human Development Index (UNDP, 2015), with an estimated that 47% of the population living below the poverty line (Federal Ministry of Health, 2010, p. 11). Despite recent declines childhood mortality rates remain comparatively high with an infant mortality rate of 41 per 1,000 live births, and a under-5 mortality rate of 59 per 1000 live births (The World Bank, 2015). Several factors inhibit further improvements in health status including malnutrition, failure to control vaccine-preventable disease, limited access to water and sanitation and low rates of health services utilization.

Benishangul-Gumuz Regional State (BGRS) in north-west Ethiopia is an underserved remote and rural region that rarely benefits from large multi-year maternal and child health programmes run by the government and international agencies. BGRS borders Sudan and South Sudan and has an estimated population of 784,345. Its transport infrastructure is weak and the majority of the population has poor access to health services.

Bambasi and Assosa districts (referred to as Woredas in Ethiopia) are lowland areas which are inhabited by predominantly Muslim agrarian communities. The average household size is 5 and the total population in both Woredas in 2015 was 169,615 (105,430 and 64,185 respectively), of which 5,208 were children <1year (3,237 and 1,971respectively).

Figure 1: Benishangul-Gumuz Regional State in Ethiopia

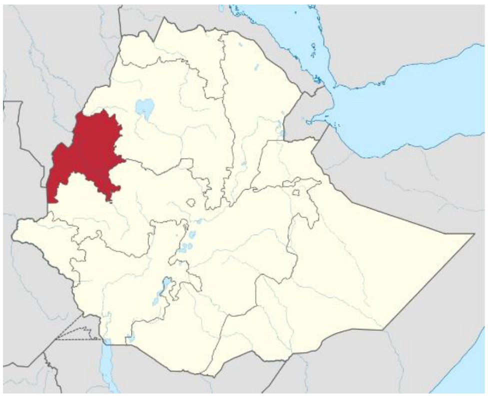

"Benishangul-Gumuz in Ethiopia" by TUBS - Own work. This vector graphics image was created with Adobe Illustrator. This file was uploaded with Commonist. This vector image includes elements that have been taken or adapted from this: Ethiopia location map.svg (by NordNordWest). Licensed under CC BY-SA 3.0 via Commons - https://commons.wikimedia.org/wiki/File:Benishangul-Gumuz_in_Ethiopia.svg#/media/File:Benishangul-Gumuz_in_Ethiopia.svg

Factors influencing vaccine uptake in the study area

A total of 13% of children living in BGRS were reported to have received no vaccines at all in the 2011 Demographic Health Survey, and coverage for Penta 3 was reported at 41.7%, which is only slightly above the national average of 37% (Central Statistical Agency [Ethiopia] and ICF International, 2012).

Based on IRC implementers field visit observations and information obtained from Woreda health officers the primary reasons for low immunisation coverage are:

- Low awareness and misconceptions about immunisation, particularly around the vaccination schedule;
- Poor health-seeking behaviours of mothers, often due to a lack of confidence in the system;
- Lack of access to immunisation services, particularly due to irregular and poorly targeted immunisation outreach services;
- Generally low capacity level among health workers, especially related to defaulter tracing and stock management; and
- Shortage of vaccines at health facilities, most often due to poor forecasting of vaccine needs and ruptures in the cold chain.

Vaccine refusal has not been observed to be an issue in the study area, although pockets of hesitancy, due to lack of convenient service, lost confidence in services, and some complacency about adherence to the childhood immunisation schedule exist.

Health system considerations

Ethiopia has a decentralized health policy, which encourages the participation of communities. To create community ownership and empowerment in the health sector, the government introduced the Health Extension Programme in 2002. The underlying vision is that if you transfer health knowledge to the community, households can take responsibility for protecting and maintaining their own health. The Health Extension Programme serves as the primary vehicle for implementation of community-centred essential health care packages, and facilitates referral processes from grass-roots to secondary and tertiary levels. The lowest level of referral system in Ethiopia is the primary health care unit (PHCU), which is composed of five satellite health posts, one health centre and one primary hospital. Health extension workers (HEWs) run the health posts where they provide preventive and basic curative services, including immunisation, and conduct household visits and immunisation outreach services at the community level.

To extend the reach of the Health Extension Programme and enhance community participation the Ethiopian government introduced a female led Health Development Army (HDA) in 2014. The aim of the HAD is to scale up and reinforce positive health practices at household level by organizing and mobilizing families. The HDA strategy involves creating a network of groups of five households, each linked to a model household led by a member of the HDA. Health extension workers based at health posts, HDA team leaders and civil service and political leaders support the HDA network. The decentralization of the health system in Ethiopia has increased decision-making powers at Woreda health system level, with the aim of improving health care delivery at community level.

At a Woreda level, there is a Woreda Health Officer (WoHO) with a responsibility of managing PHCUs. Where PHCUs are fully developed, Health Centers manage satellite Health Posts but where PHCUs are not functional such as in BGRS, the health posts directly report to the WoHOs. Each health post, usually one health post per Kebele, the smallest administrative unit in Ethiopia, is staffed with two government salaried female HEWs. The HEWs implement 16 health extension packages identified in the Health Extension Program. At community level, the HEWs select and train HDAs and HDA leaders. HDAs report to HDA leaders and the HDA leaders in turn report to the HEWs. Immunisation services are usually offered at health centers and the health posts that have a functioning cold chain once or twice per week. Normally, once per month an immunisation outreach service is scheduled in communities farther than 5km from fixed immunisation services at the center or post level. Due to resource constraints, outreach services are often conducted at the health post level; posts with or without functional cold chains.

Assosa Woreda has 74 Kebeles, 67 health posts and 3 health centers. In Bambasi Woreda there are 43 Kebeles, 41 health posts and 1 health center. In terms of human resources, there are 947 Health Development Army (HDAs) leaders, 209 Health Extension Workers (HEWs) and 91 nurses.

IRC's Community Engagement Strategy: 'The Fifth Child Project'

The IRC has been working with WoHOs, HEWs, members of the HDAs and local communities in Assosa and Bambasi Woredas since 2013. This work has resulted in the development of an integrated CE strategy and related activities, which seek to address factors that contribute to low vaccination coverage. This strategy is called 'The Fifth Child Project' and its' aim is to close the childhood immunisation gap by extending the pre-existing health infrastructure for immunisation, introducing new tools and fostering community co-management of vaccination activities.

The 'Fifth Child Project' applies an overarching and crosscutting community engagement approach, and consists of a communication and a defaulter tracing component. Essential systems support is also provided by IRC to ensure that immunisation services meet basic MoH policy standards (e.g. cold chain functionality, operational support for vaccine outreach session).

The project is informed by a theory of change which considers the feasibility of the project, describes the inputs and assumptions and incorporates three main inter-related causal pathways: i) enhanced engagement on immunisation at household and community levels, ii) improved defaulter tracing, and iii) underlying immunisation systems support. A full description and a diagram of the theory of change are provided in Appendix 1.

Cross-cutting community engagement approach

Objective:

To engender community level shared responsibility for immunisation by involving community leaders in the management of project activities in their geographic areas and introducing mechanisms for improved community ownership.

Community co-management of immunisation services and activities is facilitated by a feedback mechanism between HEWs based at health posts, HDAs and local community leaders (e.g. village, clan and religious leaders and schoolteachers). Community leaders

are responsible for supporting HEWs work with HDAs, ensuring that all pregnant women and mothers with children <1year receive a colour coded health calendars, and helping HEWs and HDAs trace vaccine defaulters and raise awareness about immunisation and related outreach events. Teachers also run regular educational sessions in schools. On a monthly basis community leaders support the planning and community mobilisation for immunisation outreach sessions. Some leaders also attend the outreach sessions in order to monitor whether: the session took place as scheduled; HEWs were available; EPI supplies and vaccines were available; and defaulting children attended. Participating leaders have told the IRC project teams that HEWs have become more accountable to communities and adherent to plans for outreach services since they began attending the sessions.

Observations from community leader outreach monitoring are brought forward in the bi-monthly Kebele command post meetings, which are attended by community leaders, HDALs, HEWs and politically appointed Kebele leaders. The HEWs present a performance overview of defaulter tracing trends (# of defaulters/month/Kebele) and HDALs and village leaders present their views on the opportunities and remaining barriers to achieving increasing immunisation coverage in their areas. Serious issues are also brought forward at quarterly Woreda-level meetings attended by Woreda Health Officers (WoHO) and Kebele leaders.

In July and October 2015 IRC ran training events for Kebele leaders and community leaders on the use of the health calendar and defaulter tracing tool and addressing immunisation related misconceptions. The purpose of the training was to create harmonization between the community leaders, Kebele leaders and HEWs and reduce any myths circulating in the community.

Project components

1. Communication & behaviour change

Objectives

To improve the quality of interactions and counselling between HEWs/HDAs and parents and caregivers of vaccine defaulter children by:

a. *Providing households with new-borns with a colour-coded health calendar – known as "Mama's Matawesha" (mother's reminder) and using this as an education tool during HEW/HDA routine and targeted home visits.*
b. *Mentoring and training HEWs & HDAs in interpersonal communication skills.*

Colour-Coded Health Calendar-Mama Matawesha

The Mama's Matawesha calendar tool is given to pregnant women during antenatal care visits or to households with new-born children and is used by HEWs and HDAs during home visits. Use of the calendars aims to help address identified barriers of caregiver understanding around the timing (and purpose) of the uptake of critical maternal and child health services. The calendar contains pictorial descriptions of five key health services, as well as warning signs of pregnancy-related dangers and is designed to reflect a continuum of care covering antenatal care, skilled delivery at birth, postnatal care, immunisation and family planning. During home visits, HEWs/HDAs describe the colour-coded images on the tool and place the same coloured stickers on appointment days to help remind families about important health post visits, including immunisation

appointments. In case of missed appointments, HDAs visit and counsel the caregivers on catch-up immunisation and will place an additional sticker on the calendar to indicate the next outreach date. The HDAs uses the calendar to initiate broader conversations with the family since it is known that women are often not the main or only decision-makers within the household with regards to the health of their children. That HDA visits the same household again a few days prior to the outreach to promote attendance. (More detail about the calendar is provided in Appendix 2)

Interpersonal communication skills training and mentorship

Training of HEWs to date has focussed on the use of the colour coded health calendars and the defaulter tracing tool. As part of this instruction HEWs have also received guidance on how to raise community awareness of immunisation and address questions and concerns in public gatherings or at household level. IRC health officers and WoHO are responsible for supporting and mentoring HEWs. IRC health officers run monthly supervision meetings and participate in quarterly Zonal HEWs supervision arranged by WoHOs. HEWs in turn are responsible for supervising and supporting HDAs. They conduct weekly meetings with HDALs leaders at health posts to review recent achievements, discuss needs for home visit related follow-up and plan activities for the coming week. HEWs also pass on any training they have received to all HDAs linked to their health post.

Plans are being finalised to deliver additional interpersonal skills and immunisation training to both HEWs and HDAs. IRC implementers are in the process of identifying and finalizing an appropriate curriculum and tools for this HEW and HAD training.

2. Tracing and follow-up of defaulting and unimmunized children

Objectives

a. *To introduce an intuitive tracing system that enables HEWs and HDAs to easily identify and follow-up vaccine defaulters.*
b. *To involve community leaders in arranging vaccination outreach sessions and encouraging vaccine defaulter attendance.*

Defaulter tracing system

The project involves the use of multiple tools, the main ones being new-born registration books (existing), immunisation registration books (existing), colour-coded health calendars (new with project), and a defaulter tracing tool (DTT) (new with project). HEWs, with support from HDAs, register all new-borns in the catchment area of the health post in new-born registration books, and then use the data from the new-born registration books to plan immunisations and to follow up-with each child to get immunized. When children come into the facility for immunisation for the first time, HEW register each child in an immunisation registration book, administer initial immunisations and calculate subsequent immunisation dates. To facilitate individual defaulter tracing, IRC and the WoHOs co-designed the DTT, and trained HEWs and HDALs on how to use it. Every time an immunisation session takes place, HEWs update the immunisation registration book, and review it to check if there were any defaulters during the session. If new-borns do not attend for immunisation HEWs and HDAs conduct home visits to counsel caregivers about the importance of vaccination and to inform them about when the next outreach will take place.

The DTT is a simple paper-based document template with three built-in carbon copies used at the health post level that records basic infant/caregiver information and antigens missed for all defaulters/village. The list of defaulters is generated from immunisation registration books. HEWs identify the list of defaulters following an outreach session and list them in the DTT by village. During *command post* meetings, the carbon copy of the DTT with the number of defaulters is presented and discussed with HEWs and HDALs. Command post meetings are formed by a team of members from different government sectors (teachers, Kebele leaders, health officers) and are established in each Kebele to review and follow-up performance of health service delivery. EPI-related corrective actions including defaulter tracing and improvement of data collection are collaboratively planned.

Copies of the DTT are given to the HDAL by HEWs so that they, in turn, inform HDAs of which defaulters to visit that month. The HDAs then conduct home visits to counsel families whose children have defaulted or never been immunised to promote catch-up immunisation. Once visits are conducted, HDAs inform HDA Leaders who both note this on the DTT and return the DTT to the HEWs at the next meeting. The second copy is shared with the Woreda Health Office and an IRC Health Officer for monitoring purposes. The third copy remains in the DTT pad for the HEW to use for follow up.

Vaccine outreach sessions jointly planned by HEWs/HDAs and community leaders

The Ministry of Health determines the number of vaccine outreach activities that will be required and can be implemented at health post level on an annual basis. It is then up to the HEWs and WoHO to arrange these outreach sessions. 'The Fifth Child Project' supports this planning by enabling HEWs and WoHO to identify areas with high numbers of vaccine defaulters and providing the infrastructure to engage with community leaders in outreach preparations. Community leaders advise on good locations and times for vaccine outreach activities, and support HDAs in community mobilisation with a specific focus on encouraging vaccine defaulters to attend.

Rationale for the formative evaluation

The 'Fifth Child Project' aims to increase vaccination coverage by working with local communities in a way that promotes the principle of shared responsibility for immunisation. Community leaders are encouraged to co-manage local immunisation activities with HEWs and members of the HDA, and parents and caregivers are given more opportunity to discuss and learn about and access immunisation services. New tools like the Mama Matawesha calendar and the DTT have been developed and introduced in order to support communication and the timely uptake of vaccinations.

After two years of implementation in 118 Kebeles across Assosa and Bambasi Woredas the Fifth Child Project has reached the stage when its approach and components need to be formally evaluated. The colour-coded calendar has been used since November 2013, and the DTT was introduced in 2015. Funds have been secured to ensure the continuation of the project across the same geographic area in 2016, which provides an opportunity to undertake a formative evaluation of the project.

The study will provide evidence relevant for policy makers and program managers involved in determining the future directions of national strategies for improving vaccination coverage in remote and vulnerable populations.

Purpose and design of the formative evaluation

The purpose of this study is to evaluate the contribution of community co-managed caregiver counselling and defaulter tracing to improving immunisation rates. 'The Fifth Child Project' consists of an overarching CE approach and two complementary communication and defaulter tracing components, hence the evaluation will assess the overall effect of these activities without aiming to tease out their individual benefits. The CE approach and component activities are dependent on each other since they constitute a response to different factors, which contribute to low and stagnating immunisation coverage in Ethiopia. The calendar seeks to increase uptake by reminding caregivers of the dates on which they need to take their children for vaccination. The defaulter tracing tool helps identify children who need to be followed up by HEWs and HDAs. Finally, the CE approach seeks to engender community level shared responsibility for immunisation by involving community leaders in the management of project activities in their areas.

Study aim

This study aims to evaluate 'The Fifth Child Project' in terms of integration into the local health system, utilisation and acceptability by the community, implementation of the CE approach and project components, and the overall effect on immunisation performance.

Study objectives

The underlying rationale of objectives 1-5 is to obtain data that will allow us to determine whether the project is being implemented as planned, to evaluate the progress of the theory of change and consider what is working well or what needs or is being changed to improve practice.

1. To observe how 'The Fifth Child Project' is integrated within the local health system and document health workers/officials, community leaders and caregiver's perspectives and experiences of this project and its' contribution;

2. To explore community leaders and health workers' views and experiences of co-managing Fifth Child Project activities;

3. To observe how the 'Mama Matawesha' tool and interpersonal communication skills training are used and applied in HDA and HEW interactions with caregivers, and document HDAs and HEWs perspectives and experiences of using the tool and applying new skills;

4. To explore caregiver's perspectives and experiences of interactions with health workers and community leaders, who co-manage Fifth Child Project activities;

5. To observe how the defaulter tracing system and tool is being applied and document health workers/officials', community leaders' and caregivers' perspectives and experiences of the system and tool;

6. To assess the performance of 'The Fifth Child Project' by reviewing defaulter statistics and monitoring trends in the uptake of routine immunisations, specifically Penta 3, the combined diphtheria, pertussis, tetanus, hepatitis B, and Haemophilus influenza type B vaccine.

More detailed evaluation questions

Cross-cutting community engagement approach

1. How is the CE approach being implemented, and how acceptable is it to community members, leaders, HEWs, HDAs and health officials?
2. What are community leaders' and health workers' experiences and perspectives on being involved in co-managing immunisation activities?
3. What are community leaders' and health workers' views on the effectiveness of the CE approach in supporting the planning and implementation of immunisation activities?
4. How well is 'The Fifth Child Project' integrated into existing health system immunisation services?
5. How could the CE approach be improved based on current experience? Have there been any innovations already developed by the health workers or suggested by the community leaders in the course of implementing the activities?

Communication & behaviour change

1. What are caregivers' and health workers' views on the colour-coded calendar and what is their experience of using it?
2. How does the colour coded health calendar support immunisation communication (including defaulter counselling) between health workers and caregivers?
3. How effective is the calendar at ensuring timely uptake of routine childhood immunisations?
4. What are caregivers' views about the quality of their interactions with health workers during home visits?
5. How do health workers apply learning gained from in interpersonal skills training and mentoring in their interactions with caregivers?
6. How could household level communication between HEWs, HDAs, or HDALs and caregivers be further improved based on current experience? Have there been any innovations already developed by the health workers or suggested by the community leaders in the course of implementing the activities?

Tracing and follow-up of defaulting and unimmunized children

1. What are health workers' views on the acceptability and functionality of the defaulter tracing system and the defaulter tracing tool?
2. How is joint health worker and community leader coordination and monitoring of immunisation outreach sessions working?
3. What is the effect of community leaders' involvement in planning, coordination and monitoring of outreach sessions and mobilising caregivers of defaulting children to attend?
4. What are caregivers' views and experience of community leaders' involvement in defaulter tracing activities?
5. How could the defaulter tracing system, its constituent tools and the joint planning and implementation of outreach sessions be improved? Have there been any innovations already developed by the health workers or suggested by the community leaders in the course of implementing the activities?

Study Design

This formative evaluation will use a primarily qualitative research approach, which will be complemented by the quantitative analysis of routine immunisation and project data. The study design is post-test since the project has already started and the evaluation and related measurements will occur after its introduction. A variety of data collection methods will be used to assess the following: 1) acceptability and utilisation of the integrated CE approach among health workers, community leaders, and caregivers - semi-structured interviews, focus group discussions and observations, (3) quality of health worker interactions with caregivers and use of the calendar – interviews with caregivers, HEWs and HDAs, observations and quality audits, (2) performance of the defaulter tracing system - review of immunisation data, observations and quality audits, (3) overall performance of 'The Fifth Child Project'- examining trends in DPT3 coverage from the Ministry of Health information system.

The formative evaluation will span a time-period of 1 year (see Timeline) which will support the monitoring of trends in immunisation uptake and allow us to conduct two sequences of qualitative data collection, the latter being informed by the analysis of initial data.

Evaluation team

The evaluation team comprises of investigators from both partner organisations and their responsibilities are outlined on page 3. LSHTM investigators will provide leadership in terms of evaluation design, data analysis and write up and Ethiopia based investigators will monitor routine project an immunisation data and oversee the implementation of qualitative evaluation activities on the ground. The Ethiopian investigators will also be responsible for recruiting external research assistants (RAs) who will conduct the interviews, focus groups, observations and quality audits.

An evaluation training workshop will be organised in April 2016 and take place at IRC facilities in BGRS. LSHTM investigators will facilitate the workshop with the support of Ethiopian team members. The purpose of this interactive workshop is to provide a detailed overview of the methods that will be used to collect qualitative data, and provide the RAs with the skills and tools they need to obtain and support the analysis of this data. The LSHTM investigators will also observe a small number of interviews, observational activities and quality audits to mentor the research assistants and support high quality data collection.

Sampling Frame

'The Fifth Child Project' was iteratively developed and implemented in 114 Kebeles (smallest administrative unit in Ethiopia e.g. neighbourhood) across Assosa and Bambasi Woredas between November 2013-December 2015. This involved working with 929 HDA/HDALs, 216 HEWs (from 105 health posts), and many community leaders to increase the uptake of routine immunisations in a cohort of 5,002 infants. Funding has been secured to cover the same programming and geographic scope in 2016, however only three Kebeles will feature in the formative evaluation. These Kebeles were chosen to represent areas that are situated at different distances from the Woreda main town and that differ in terms of population size. Two of the Kebeles are also mainly inhabited by indigenous Muslim populations whereas the other is home to Christian settler groups, who moved to BGRS from other parts of Ethiopia. Agriculture is the main income generating activity across all 3 of these Kebeles with some mining supplementing this in Mugfude. Table 1

provides more details about these Kebeles; please note that we estimate that there are about 60 community leaders across these Kebeles.

Table 1: Composition of selected study sites

Kebeles (Woreda) for formative evaluation	Distance from Woreda main town	HEWs/ Health post	HDAs/HDA leaders	Total population	Population <1year (infants)
Mugfude (Assosa)	58km	2 / 1	42 / 14	2,011	62
Amba 17 (Assosa)	32km	2 / 1	18 / 15	1,035	32
Jematsa (Bambasi)	24km	2 / 1	43 / 11	1,432	46
**Total**		**6 / 3**	**103 / 40**	**4,478**	**120**

Methods

System for monitoring immunisation and project data

The aim of this system is to ensure that immunisation and project data are collected in a systematic way that will allow us to assess the overall performance of 'The Fifth Child Project'.

Data collection procedures

There is an immunisation registration book in each health post, and the HEWs register daily activities in this registration book. On a monthly basis, HEWs compile a report and share this with the WoHO and IRC health officers and fill-in the DTT with the list of defaulters for that month as well as any carry over from the previous month. The IRC health officers submit this data to the health manager who is based at the field office, and the manager enters the data into an excel database and shares it with the Addis Ababa health unit. The IRC staff also record information collected from performance review meetings, training and supervision events. The information includes number of participants, key challenges faced by the HEWs and HDA/HDAL, major successes, and lessons that can be shared. The project team also utilises the DTT data to identify number of defaulter children and those that are traced and immunized.

Data collected

The data collected includes the number of children vaccinated, the number of outreach sessions conducted, the number of colour coded health calendars distributed, the number of defaulting children identified and vaccinated, the number of home visits conducted and information about any other key activities that have taken place during that month, such as training, review meetings, supervision group meetings and accompanied supervision home visits.

Key indicators

The following key indicators will be specifically tracked: a) Penta3 coverage in each target Kebele, b) The number of infant defaulters who are identified and the number of these who are subsequently vaccinated, c) The number of outreach sessions conducted, (as compared to the number planned) and the total number of infants and sub-total number of defaulters vaccinated during outreaches.

Qualitative interviews and focus groups

The qualitative interviews and focus groups will be conducted by the research assistants in two stages and the LSHTM investigators may also attend and support this data collection. The first stage will take place in May-June 2016 immediately after the evaluation methods training workshop. The plan is to conduct a total of 54 semi-structured interviews: 12 with caregivers, 9 with community leaders, 3 with nurses, 6 with HEWs, 3 with HDALs, 6 with HDAs, 6 with health centre leaders, 3 with HEW supervisors, 4 with Woreda health officials, and 2 with EPI coordinators. In addition, 3 focus groups will be conducted with caregivers (2 with mothers, 1 with fathers) and 3 with HDAs. We will group the participants as follows: Health care workers (health centre leaders, nurses, HEWs=15), Community members (community leaders=9), Parents/caregivers (parents/caregivers=approximate 36 depending on size of focus groups), Community health workers (HDAs/HDALs=approximated 33 depending on size of focus groups) & Woreda staff (Health officials, EPI coordinators, HEW supervisors=9)). We will group the participants as follows: Health care workers (health centre leaders, nurses, HEWs), Community members (parents/caregivers/community leaders), Community health workers (HDAs/HDALs) & Woreda staff (Health officials, EPI coordinators, HEW supervisors)). The subsequent analysis of the data collected, ongoing observations and experience gained during the implementation of the project will inform the second stage of data collection that is planned for September 2016. During this second stage some participants may be asked to take part in a second interview and additional interviewees may be recruited to explore and address issues that have arisen in the course of the implementation of the project or in the analysis of interview and observational data that has already been collected.

We have chosen to adopt a semi-structured approach for the interviews and focus groups. This will allow the RAs to cover predefined topics and provide the necessary flexibility for the interview to be shaped by interviewees' interests, their roles and experiences. The semi-structured interviews with HEWs and HDAs/HDALs and the focus group with HDAs will explore the acceptability and utilisation of the CE approach, the colour coded health calendar, the DTT and tracing system. The semi-structured interviews with community leaders explore the acceptability and utilisation of the CE approach and related co-management of immunisation activities. The focus groups and semi-structured interviews with caregivers will focus on their experience of interactions with HEWs and HDAs, the acceptability and their use of the colour-coded calendars, and their views about the CE approach and defaulter tracing activities, including vaccination outreaches. We will develop topic guides that the RAs can use to structure the exchanges with caregivers, health workers and community leaders. These topic guides will be finalised during the evaluation training workshop in April 2016 and will be informed by the evaluation questions cited on page 12. They will be translated into Amharic and the local language, Rutegna, which is spoken in the 3 Kebeles where the evaluation is taking place.

The interviews and focus groups will be conducted by two teams consisting of 2 RAs and one RA supervisor. In the focus groups one RA will facilitate the discussion and conversation and the other will take notes and observe non-verbal communication. The interviews and focus groups will be conducted in participant's local language and will last approximately 1-1.5 hours. The RAs will be recruited based on their knowledge of the local language and will transcribe and translate the interviews. With participants' permission we will audio record the interviews and focus groups with the use of a digital recorder. In cases where people taking part in individual interviewees would prefer not to be recorded, the RAs will take field notes during and after the interview. If individual focus group participants do not want to be recorded the RAs will invite them to take part in an individual interview. At the end of the interviews and focus groups the RAs will

compile field notes summarising the interaction paying particular attention to the context, quality and feel of the exchange, content interviewees emphasised and content that needs specific follow up. Basic socio-demographic information (age, gender, religion, occupation, address, place of work, relevant background experience in health and how long they have been doing current job for health workers, age of child, their immunisation status, other family members for caregivers) will be collected at the start of the interview.

Participant recruitment

Potential participants will be approached by the RAs and be provided with a study information letter (see Appendix 3) that will be translated into Amharic and the relevant local languages. The information letter will explain what the study is, and what will happen with the results. It will also explain that all data that is collected as part of evaluation activities will be anonymised before it is shared. During this first encounter RAs will also provide potential participants with a brief verbal summary of the study and ask them whether they might be willing to take part in an interview or focus group. Interview appointments will be arranged for those who express interest. The RAs will assure these potential participants that they can change their mind prior to an interview/focus group taking place and will leave them their contact details. At the interview/focus group appointments the RAs will read through the study information sheet with all potential participants giving them the opportunity to ask questions. After doing this each person will be asked on a one to one basis whether they are willing to take part in the study. They will be reassured that they can withdraw from the study at any point and will be given the RAs contact details. Verbal consent is the preferred practice for obtaining agreement for participating in this type of health research in this region. Asking people to sign forms is generally viewed as a sensitive practice that can evoke fear about what participants are being asked to do or provide, rather than being a means of protecting their rights. Care will be taken to ensure that the consent process is conducted in a private and confidential manner and no record will be kept of individuals who chose not to participate. Participants willingness to take part in this research will be documented by asking each participant to record a statement before an interview/focus groups takes place. In this statement they will be asked to cite their name and the date and state that they are willing to take part in the research and understand that taking part in the study is voluntary, that they are free to withdraw at any time and that the give up no rights by consenting. These recordings will be stored securely on password protected computers at the IRC offices, separately from the recordings and transcripts of the actual interviews or focus groups.

Participant sampling

Health officers – This group includes two Woreda health official and two EPI coordinators (Assosa and Bambasi). It also includes three HEW supervisors and three health centre leaders (one per Kebele).

HEWs – All of the six HEWs working at the health posts of the selected Kebeles will be invited to participate in an individual semi-structured interview. The HEWs will be approached at their respective health posts and the interviews will take place at the health posts or the HEWs home outside of working hours. Nurses are also currently based at the kebele health posts; we will seek to recruit all nurses based in our sites of interest.

HDAs – There are currently 103 HDAs across all three Kebeles and 40 HDA leaders. We will aim to interview thee HDAs and one HDA leader per Kebele, aiming for a total of nine

HDAs and thee HDA leaders. We will also conduct three focus groups with HDAs, one in each Kebele. The HDAs and HDA leaders will be purposively selected in a way that ensures maximum variation in terms of age, villages, and previous health work experience (HDA members are all female). The project team will obtain guidance from the HEWs to select the HDAs and HDA leaders. The interviews with HDAs and HDALs will take place at their homes or a private place of their choosing.

Community leaders – There are approximately 60 community leaders across the three Kebeles. We will aim to interview three community leaders per Kebele, aiming for a total of nine community leaders. Community leaders will be selected purposively in a way that ensures maximum variation in terms of gender, age, religion, villages and level of involvement in 'The Fifth Child Project'. The research assistants will seek guidance from the HEWs in the identification and selection of community leaders. The interviews will be conducted in community leader's homes or private place of their choosing.

Caregivers – There are approximately 120 infants in all three Kebeles. We will aim to interview 12 caregivers (four per Kebele; two mothers, one father, one mother in law or significant other family member) and organise three focus groups with 8-10 participants each (two for mothers, one for fathers). Potential participants will be selected purposively in a way that ensures maximum variation in terms of gender, age, religion, and villages. The interviews will be conducted in caregiver's homes. Given the setting the caregiver interviews may involve more than one person depending on who is present at the time of the interview. To be sensitive to any privacy concerns, the main interviewee will be offered the option of seeking a more private place or rescheduling if they would prefer, if they do not mind the interview will go ahead in the home and the RA will obtain verbal consent from other key people present. The focus groups will be conducted in a community building like a church or school that is private and where participants will feel free to speak openly about their views and experiences. Consideration will be given to providing and supporting appropriate childcare arrangements during the focus group.

Participant observation of project activities

Participant observation of project activities will take place primarily during April-June 2016 when the RAs and the LSHTM investigators will participate in and observe a variety of project activities which are summarised below. They will seek approval from the relevant leaders/participants to be present during these activities and inform all participants why they are observing and what they will do with the information they collect. Where appropriate the RAs will make notes during the events they observe. These notes and their recollections of each event will be written up as soon as possible after the event has taken place. These notes will describe the type of event that was observed, the type and number of people present, what took place and what was discussed. The notes will be anonymised and codes will be used to indicate where the meeting took place (e.g. Kebele 1). Some observations will occur during the course of the study in order to observe events that may not occur during May-June 2016 e.g. vaccine defaulter visits, vaccine outreaches and routine first visits to new babies. These events will be observed by IRC staff who are not involved in 'The Fifth Child Project' but who are based in the area and speak the local language. They will receive training in these evaluation activities as part of the workshop run by LSHTM investigators at the end of April 2016. Some observations may also take place during the second round of interviews in September 2016.

1. Activities linked to routine immunisation to which 'The Fifth Child Project' contributes

- Review/supervision meetings between HEWs, IRC HOs and WoHOs
- Review and supervision meetings between HEWs, HDALs and HDAs
- Bi-monthly Kebele command post meetings attended by community leaders, HDALs, HEWs and politically appointed Kebele leaders.
- Quarterly Woreda-level meetings attended by WoHOs and Kebele leaders.

The purpose of these observations is to learn about and record any challenges faced by health workers in carrying out and managing immunisation activities, noting also how these are addressed. The RAs will also seek to document how 'The Fifth Child Project' is integrated into these routine events and how community leaders are involved.

2. *Training activities*

- Interpersonal skills and immunisation training run by IRC for HEWs
- Interpersonal skills and immunisation training run by HDALs/HDAs by HEWs with support from IRC
- Other training events organized by the Ministry of Health

The purpose of these observations is to gain insights into the skills that health workers are taught and to learn from any experiences/scenarios shared by HDAs/HEWs during the training.

3. *Defaulter tracing activities including vaccine outreach events*

- HEWs, HDALs/HDAs use of the DTT as part of immunisation activities
- HEWs, HDALs/HDAs and community leaders planning for vaccine outreaches and defaulter follow up
- Community mobilisation and defaulter follow-up conducted by HDAs and community leaders prior to vaccine outreaches
- Health Post linked immunisation outreach events

The purpose of these observations is to gain insights into how the HEWs/HDAs use the DTT, noting what works well and what is difficult. These observations will also help RAs gain insights into how community co-management of defaulter tracing activities and vaccine outreach sessions is working. Attention will be paid to the rapport/dynamic between HEWs/HDAs and community leaders, the level joint ownership in planning and the nature of interactions between HEWs-HDAs/community leaders and community members, specifically vaccine defaulters, and how community mobilisation is done.

4. HEW and HDA home visits

This observational work will take place after the HEWs and HDALs/HDAs have received the additional interpersonal skills training scheduled to take place in April 2016. The training curriculum and key learning imparted will inform the focus of the interviews and an observation sheet will be developed for these accompanied visits following the training. This sheet will allow the RAs to assess different aspects of the visit based on pre-determined measurement criteria; purpose of the visit according to the HEW/HDA, initial greeting, how rapport is established, who is involved in the visit, who is present, whether and how the colour-coded health calendar is used, what key immunisation messages or other health messages are imparted, what caregivers say or ask, the nature and quality of interactions between the caregiver and the HEWs/HDAs (behaviour, mannerisms, active listening), and actions agreed.

The plan is to conduct approximately 15 accompanied home visits across the three Kebeles. They will include visits to homes with new-born infants, defaulting infants and regular home visits conducted by HDAs. The HEWs and HDAs/HDALs will be informed about these accompanied home visits during the training. They will be advised that this is a way to evaluate the effectiveness of the training and the use colour-coded health calendars and not a performance management exercise. The RAs will share their observations with the people they accompany but will not provide feedback to their line mangers. The HEWs and HDALs/HDAs will be able to opt out from this observational exercise if they feel uncomfortable with this process. Their verbal consent will be obtained in the same way as described for the interviews and focus groups. The RAs will conduct home visit with all of the HEWs and a randomly selected sample of HDALs/HDAs. The HEWs and HDALs/HDAs will inform the relevant households in advance that an additional person will join their visits to ensure that the caregiver and other family members provide their permission for the RA to join the home visit.

Data management and analysis

Immunisation and project data

Routine immunisation and project data will be stored in an excel database on password-protected computers. The use of basic descriptive statistics techniques will allow us to observe trends in vaccination coverages and detect changes in the rates of vaccine defaulters over the 12 months of implementation. The descriptive statistics include calculating frequencies to determine the coverage of the different antigens. The project team will compile graphs using this data to observe trends and take corrective actions when needed.

Interview, focus group and observational data

Data will consist of audio files, related transcripts and fieldnotes from each interview and focus groups, and copies of observational event fieldnotes and home visit reviews. The fieldnote summaries will be written in English and the audio files will be directly transcribed and then translated with the support of the hired interpreters. This data will be stored electronically on password protected computers. Each participant will be allocated a numerical identifier (e.g. Community Member #1, Parent/caregiver #3, Health Care Worker #4, Community Health Worker #1, Woreda Staff #5) and only the Ethiopian investigators, the RAs and LSHTM investigators will have access to the socio-demographic sheet that will link the location code and numerical identifier to particular interviewee. The electronic audio files and transcripts will only refer to the code and numerical identifier and these documents will be stored separately to the excel spreadsheet listing participants' details. Participant's socio-demographic information sheets will be stored in a locked file at IRC offices, which only the RAs, the LSHTM investigators and Ethiopian investigators will be able to access. Interview transcripts, field notes, scanned copies of participant's socio-demographic information and routine immunisation and project data will be sent to LSHTM investigators by email as password-protected zip file attachments. These files will be stored on the secure LSHTM network and will only be accessed by the LSHTM study investigators. The emails will be destroyed as soon as the files have been retrieved.

The data analysis will be led by the LSHTM investigators. The RAs will send the anonymised transcripts, observational fieldnotes and quality review sheets to these investigators in password protected zipped files after they have checked the interview

transcription and translation for accuracy. The transcripts, fieldnotes and review quality sheets will be emailed sequentially as they are completed. The LSHTM investigators will download this material into a qualitative data analysis software programme (NVivo). This programme facilitates the display, coding and management of qualitative data.

The approach to data analysis will be mainly thematic (Boyatizis, 1998), although techniques outlined by Strauss and Corbin (1998), which are common to grounded theory, will also be applied i.e. open coding and the constant comparative method. Data analysis will proceed in tandem with data collection and the LSHTM investigators will meet regularly to discuss emerging findings, define codes and higher level themes and categories, and then map and finalise a coding framework. They will also have 1-2 weekly Skype calls with the RAs to discuss the analysis and fine tune interview questions where necessary. Two of the investigators (TC and EK) will both code the first three transcripts from each group (caregivers, HEWs, HDAs/HDALs, and community leaders) individually in order to be able to compare their findings, enhance consistency and start to develop a coding framework. They will then use this framework to code the next three transcripts from each group. At this point, they will meet with the LSTHM Principal Investigator (HL) to discuss the emerging findings and produce a comprehensive and coherent coding framework. They will then code the rest of the transcripts and field notes. Aspects of home visit review sheets will be analysed separately but relevant descriptive content will also be included in the overall qualitative data analysis. When this is complete, the investigators (TC, EK, HL) and RAs will meet again to summarise their preliminary findings, and decide on who they need to approach in the second round of interviews. The preliminary analysis will be shared and discussed with all research partners before the second round of interviews proceeds in September 2016. The data from this round will be processed and analysed as described above. The coding framework will be amended as necessary the final analysis will be complete by the end of October 2016 and the findings compiled and prepared for dissemination in November and December 2016.

Ethical considerations

We do not foresee any significant ethical issues since participation in this study constitutes very low risk. The information obtained from the study will also support improvement in vaccination services, which will benefit caregivers, community leaders and health workers. The RAs and LSHTM investigators who will be involved in most of the data collection do not have any professional ties with 'The Fifth Child Project'; this positioning and independence is of importance in terms of creating rapport and promoting candid and confidential exchange. The RAs and where relevant the LSHTM investigators will obtain verbal consent from participants and will ensure that their anonymity is maintained. According to Ethiopian team members, verbal consent is usual practice for this type of research is this region. They advised us that obtaining written informed consent could represent a barrier since signing forms is viewed as sensitive and provokes fear. Participants willingness to take part in this research will be documented by asking each participant to record a statement before an interview/focus groups takes place. In this statement they will be asked to cite their name and the data and state that they are willing to take part in the research and understand that taking part in the study is voluntary and that they are free to withdraw at any time. These recordings will be stored securely on password protected computers at the IRC offices.

Recordings and transcripts of interviews will be stored anonymously using a numerical identifier on password-protected computers. Only the investigators will have access to the files that link a numerical identifier to a participant's name. Each Kebele will be allocated a numerical identifier. Anonymised quotations from participant interviews may be used in study reports or published articles. Confidentiality will be maintained by referring to quotations using the code assigned to the participant's role only (e.g. Community Member 2) and extra care will be taken to ensure that participants or organisations cannot be identified through contextual information. The interviews will be audio-recorded, with the participants' consent and transcribed. Some direct quotes from the interviews may be used in reports and publications, but these will not be attributed to identifiable individuals.

Ethical approval will be sought from the LSHTM ethics committee and the Regional Health Bureau of Benishangul Gumuz Regional State.

Dissemination and reporting of results

Anticipated products or inventions resulting from the study and their use

The IRC anticipates high level of acceptance, utilization and performance of the colour coded health calendar and defaulter tracing tool. The IRC will extensively disseminate the study findings among internal and external stakeholders, and the study team has developed a stakeholder engagement and evidence uptake plan to support this work.

Disseminating results to stakeholders and the public

IRC will utilize different approaches to disseminate the study findings. This will include the use of IRC website, print materials such as formal report, brochures and leaflets, and disseminate the results through presentations. The IRC will organize formal dissemination workshops at the two operational Woredas and at regional level. The IRC team will also utilize existing forums at the national level including at the Federal Ministry of Health and Ethiopian Civil Society Health Forum. The findings will also be presented at relevant academic conferences and published in appropriate journals.

Timeline

**Timeline**	**A formative evaluation of *'The Fifth Child project'*: A new initiative to close the childhood immunisation gap in Benishangul-Gumuz Regional State in Ethiopia**											
**Events**	**01/16**	**02/16**	**03/16**	**04/16**	**05/16**	**06/16**	**07/16**	**08/16**	**09/16**	**10/16**	**11/16**	**12/16**
Stakeholder meeting												
Ethics approval obtained												
Online journal updated for lessons learnt												
Polio campaign in the area												
Recruiting & training of research assistants												
Training of research assistants and Regional Health Bureau on research methodology												
Interpersonal skills and immunisation training for HEWs and HDAs												
Collection and analysis of routine data												
Qualitative data collection #1												
Qualitative data analysis #1												
Height of rainy season + Eid al Fatal/Ramadan												
Observation and quality audits												
Observation and quality audits analysis												
National holidays 9/12, 9/24, 9/28												
Qualitative data collection #2												
Qualitative data analysis #2												
Dissemination and reporting of results												
**Main partner responsible**		Both partners			IRC			LSHTM			Notable event	

-

A formative evaluation of 'The Fifth Child Project', version 2, dated 29/02/16
LSHTM Ethics Reference 10542, Beningshangul Gumuz Regional Health Bureau Ethics Committee Reference: 674/ቢሮ m-o1

References

BOYATIZIS, R. E. 1998. *Transforming qualitative information: Thematic analysis and code development,* Lodnon, Sage.
CENTRAL STATISTICAL AGENCY [ETHIOPIA] & ICF INTERNATIONAL 2012. Ethiopian Demographic and Health Survey 2011. Addis Ababa, Ethiopia and Calverton, Maryland, USA: Central Statistical Agency and ICF International.
FAVIN, M., STEINGLASS, R., FIELDS, R., BANERJEE, K. & SAWHNEY, M. 2012. Why children are not vaccinated: a review of the grey literature. *International Health,* 4, 229-238.
FEDERAL MINISTRY OF HEALTH 2010. Ethiopia National Expanded Programme on Immunization: Comrephensive Multi-Year Plan 2011-2015. Addis Ababa
JAIN, M., TANEJA, G., AMIN, R., STEINGLASS, R. & FAVIN, M. 2015. Engaging Communities With a Simple Tool to Help Increase Immunization Coverage. *Global Health: Science and Practice,* 3, 117-125.
OWAIS, A., HANIF, B., SIDDIQUI, A., AGHA, A. & ZAIDI, A. 2011. Does improving maternal knowledge of vaccines impact infant immunization rates? A community-based randomized-controlled trial in Karachi, Pakistan. *BMC Public Health,* 11, 239.
RAINEY, J. J., WATKINS, M., RYMAN, T. K., SANDHU, P., BO, A. & BANERJEE, K. 2011. Reasons related to non-vaccination and under-vaccination of children in low and middle income countries: Findings from a systematic review of the published literature, 1999–2009. *Vaccine,* 29, 8215-8221.
RYMAN, T. K., TRAKROO, A., WALLACE, A., GUPTA, S. K., WILKINS, K., MEHTA, P. & DIETZ, V. 2011. Implementation and evaluation of the Reaching Every District (RED) strategy in Assam, India, 2005–2008. *Vaccine,* 29, 2555-2560.
SABARWAL, S., BHATIA, R., DHODY, B., PERUMAL, S., WHITE, H. & PURI, J. 2015. Engaging communities for increasing immunisation coverage: what do we know? 3ie scoping paper 3. New Dehli: International Initiative for Impact Evaluation (3ie).
STRAUSS, A. & CORBIN, J. 1998. *Basics of Qualitative Research,* London, Sage.
STREEFLAND, P., CHOWDHURY, A. M. R. & RAMOS-JIMENEZ, P. 1999. Patterns of vaccination acceptance. *Social Science and Medicine,* 49, 17051716.
THE WORLD BANK. 2015. *Mortality Rates (Accessed 2nd December 2015 at http://data.worldbank.org/indicator/SH.DYN.MORT/countries)* [Online].
UNDP 2015. Human Development Report 2013. The Rise of the South: Human Progress in a Diverse World, Ethiopia (Acccessed 2nd Dec 2015 at [http://hdr.undp.org/sites/default/files/Country-Profiles/ETH.pdf)](http://hdr.undp.org/sites/default/files/Country-Profiles/ETH.pdf).
USAID 2015. Ethiopia Factsheet (accessed 2nd Dec 2015 at [https://eads.usaid.gov/esdb/analysis/fact_sheets/ethiopia.pd)](https://eads.usaid.gov/esdb/analysis/fact_sheets/ethiopia.pd).
USMAN, H. R., RAHBAR, M. H., KRISTENSEN, S., VERMUND, S. H., KIRBY, R. S., HABIB, F. & CHAMOT, E. 2011. Randomized controlled trial to improve childhood immunization adherence in rural Pakistan: redesigned immunization card and maternal education. *Tropical Medicine & International Health,* 16, 334-342.
WHO 2013. Global vaccine action plan 2011-2020. Geneva: World Health Organization.
WHO 2015. National Immunization Coverage Scorecards Estimates for 2014. Geneva World Health Organization.

Appendix 1

Project implementation feasibility and the underlying theory of change

The implementation of 'The Fifth Child Project' is feasible since it was designed to be integrated into the existing health system and is adapted to the context and structures of maternal and child health programming in the study sites. The approach is in line with the Health Extension and Health Development Army strategies that aim to extend more services to the community level and task-shift health promotion activities to the recently trained cadre of HDAs.

Project implementers considered that any perceived additional responsibilities introduced under the intervention may contribute to HEW or HDA de-motivation and may reduce performance in other areas of community work. Given that each HDA is responsible for five households within their village, HDAs involved in this project will conduct, on average, one defaulter counselling or outreach reminder home visit per month. Neither the HDAs nor the HEWs have reported during routine IRC project monitoring and support visits that the intervention components have overburdened them.

HEWs have the highest authority within the health system in their respective Kebeles (health post catchment area) with regard to health extension services. They are also responsible for selecting HDAs, in a participatory process, and training them on selected health extension packages. HDAs are expected to serve as role models in practicing the health extension packages in their communities and to support households in their network to practice the packages. Whenever the HEWs conduct a home visit (likely only the first visit to introduce the calendar tool and the first post-natal visit) the HDA will also be present to observe and learn from the HEW in order to reinforce the messages and behaviours discussed after the HEW has left. There is a clearly delineated relationship between HEWs and HDAs, with HEWs being trainers and mentors for HDAs. The project team therefore does not anticipate any issues related to power dynamics between these two cadres.

It is important to note that HEWs and HDAs are already responsible for tracing immunization defaulters and conducting community mobilization on immunization services; however, currently they do not have appropriate tools to implement these activities. IRC introduced the tools to facilitate work conducted by HEWs and HDAs and improve their performance. The tools fully align with the work of HEWs and have so far been well received by HEWs, HDAs, Woreda Health Offices and Zonal Health Department and have not been seen as a burden to date. The contextual understanding and social and political capital established by the project team in 15 years of collaboration with key stakeholders in intervention areas, also contributes to the implementation feasibility of the program. Nevertheless, the formative evaluation aims to identify any implementation challenges as well as potential intervention iterations in order to adapt activities for increased feasibility as needed.

The theory of change incorporates three main inter-related causal pathways: improved engagement on immunization at the household and community levels, the defaulter tracing system, and underlying immunization systems support.

Household and community engagement: One of the key reasons for low immunization uptake is low level on the demand side. Key barriers to immunization include low level of awareness on the benefits of immunization as well as misconceptions about vaccines, lack of knowledge on immunization schedules, and generally poor levels of health-seeking behaviour. The colour-coded health calendar serves to address these demand-side barriers. In addition to helping caretakers keep track of immunization schedules, it also serves as a visual aid to facilitate communication between HEWs/HDAs and the household and within the household, since the calendar has pictorial descriptions on immunization and other maternal health services.

The way the calendar is used also aims to address the issue that women are often not the main or only decision-makers within the household related to the health of their children. The initial introduction of the calendar tool within the household by HEWs includes other decision-makers such as husbands and mothers-in-law so that the importance of timely immunization uptake is presented in front of the key family members allowing them to ask questions, discuss the schedule and promotion messages and come to decisions together.

The *Fifth Child* intervention is built upon the principle that strong interpersonal communication between health providers and clients can contribute to behaviour change and the increased uptake of preventive services. It anticipates that if HDAs/HEWs receive necessary training, tools (DTT and *Mama's Matawesha*) and support, they will be motivated by this principle and the connection to their communities in order to conduct effective home visits. In turn, this level of HEW/HDA dedicated will encourage caregivers to bring infants for timely immunizations.

Based on the experience of over 15 months of full project implementation, the evaluation team postulates that formative findings will demonstrate that the *Mama's Matawesha* tool simultaneously serves as an information, education and communication job aid during home visits (for HEWs and HDAs), as a reminder on fixed appointments for health services (for families) and stimulates dialogue between HEWs/HDA and their clients.

A critical component of this causal pathway is as follows: HDAs live in their communities and as such, are already trusted and familiar to their communities. However, they lack the technical tools for routine monitoring of vaccination status (i.e., the DTT) and for targeted counselling and reminders when necessary (i.e., the *Mama's Matawesha)*. To facilitate these actions, CE approaches are needed from multiple levels. During the existing bi-weekly meetings, HEWs now present the numbers of defaulters, infants due for immunization as evidence of actions needed, and mobilize community leaders to conduct outreach and find defaulters. This adds greater accountability at the community level and leads to improved targeting of service delivery.

Defaulter tracing tool: Before this intervention, the registration of pregnant women, allowing HEWs/HDAs to track mother/new-born pairs, was weak. To establish an accurate denominator of vaccine-eligible children, the intervention systematizes this registration and improves the quality of data in the child EPI register. If immunisation status data for the infant is not reflected in the register, then the HEW will review the child's vaccination card at their next health post or home visit.

The DDT causal pathway anticipates that if HEWs, HDAs and community leaders have individualised data on defaulters, they will become motivated and supportive co-managers of routine defaulter tracing/defaulter mobilisation for outreaches, aiming to protect their communities against vaccine-preventable disease.

Immunisation systems support: Sustained delivery of immunisation services by the MoH and HEP (with some support from IRC) is a precondition for the formative evaluation of this intervention. To help ensure that EPI systems remain functional, the IRC fills identified gaps related to vaccine transport, basic EPI training for HEWs, financing of outreaches and supportive supervision by WoHOs as well as cold chain functionality.

An intended outcome of this intervention is that increasing demand for EPI services could lead to uptake of other maternal and child health services at the health posts or at the higher-level health centres. For example, home visits may identify and refer more sick children, sick new-borns in particular, for PNC services or integrated management of neonatal and childhood illnesses as well as women who should access ANC and FP services.

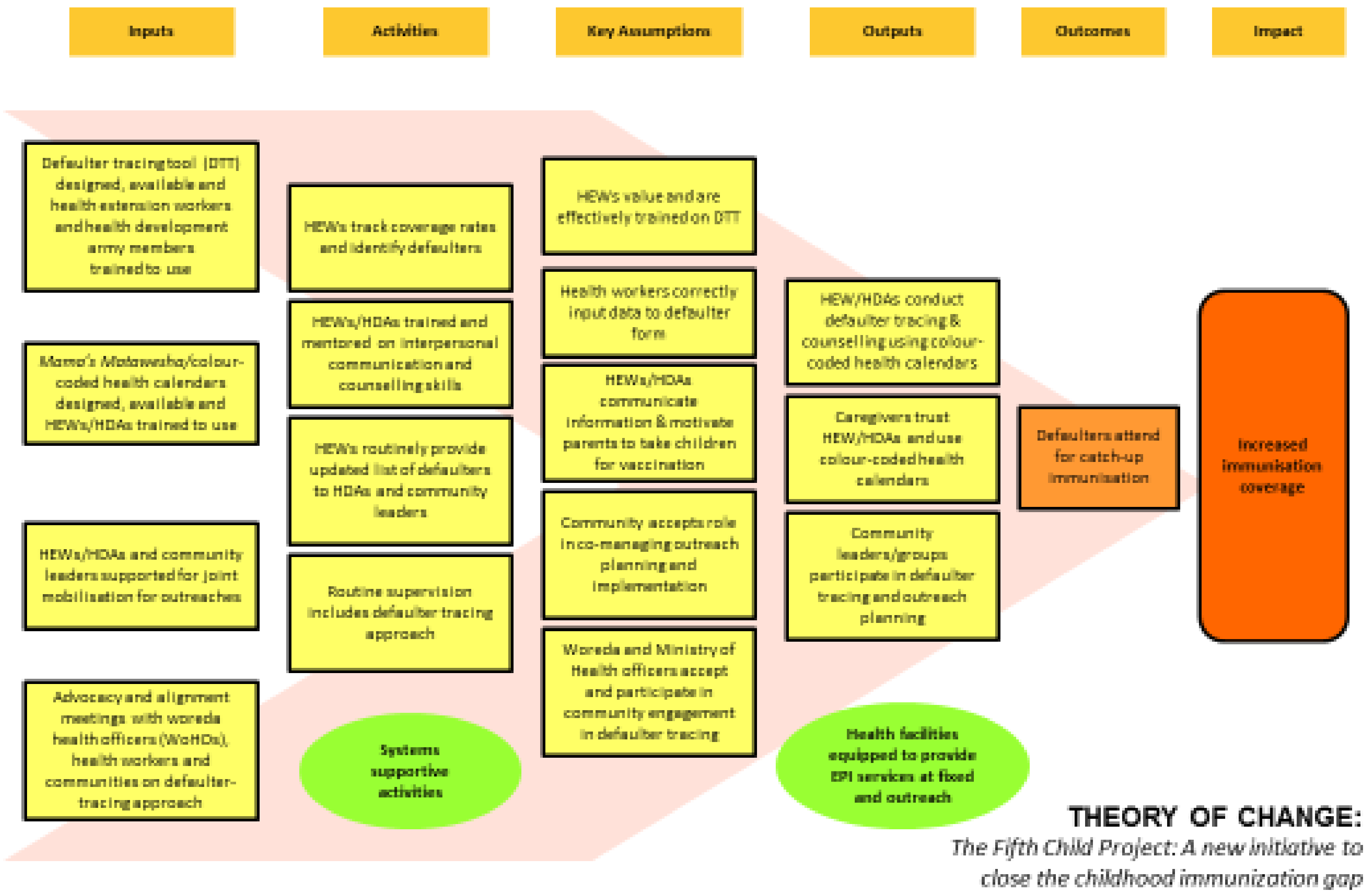
Inputs
Activities
Key Assumptions
Outputs
Outcomes
Impact
Defaulter tracing tool (DTT) designed, available and health extension workers and health development army members trained to use
Mama's Malawesha/colour-coded health calendars designed, available and HEWs/HDAs trained to use
HEWs/HDAs and community leaders supported for joint mobilisation for outreaches
Advocacy and alignment meetings with woreda health officers (WoHOs), health workers and communities on defaulter-tracing approach
HEWs track coverage rates and identify defaulters
HEWs/HDAs trained and mentored on interpersonal communication and counselling skills
HEWs routinely provide updated list of defaulters to HDAs and community leaders
Routine supervision includes defaulter tracing approach
Systems supportive activities
HEWs value and are effectively trained on DTT
Health workers correctly input data to defaulter form
HEWs/HDAs communicate information & motivate parents to take children for vaccination
Community accepts role in co-managing outreach planning and implementation
Woreda and Ministry of Health officers accept and participate in community engagement in defaulter tracing
HEW/HDAs conduct defaulter tracing & counselling using colour-coded health calendars
Caregivers trust HEW/HDAs and use colour-coded health calendars
Community leaders/groups participate in defaulter tracing and outreach planning
Health facilities equipped to provide EPI services at fixed and outreach
Defaulters attend for catch-up immunisation
Increased immunisation coverage
THEORY OF CHANGE:
The Fifth Child Project: A new initiative to close the childhood immunization gap

Appendix 2

The Colour Coded Health Calendar – 'Mama Matawesha'

The *Mama's Matawesha* colour coded health calendar is distributed to all households with pregnant women in the IRC Fifth Project catchment area and used by HEWs and HDAs during home visits. It aims to address identified barriers of caregiver understanding around the timing and purpose of the uptake of critical maternal and child health services. The calendar contains pictorial descriptions of five key health services, as well as warning signs of pregnancy-related dangers and is designed to reflect a continuum of care covering antenatal care, skilled attendant service, postnatal care, immunization and family planning. The calendar contains pictorial descriptions of the various health services, which serve as visual aid to assist mothers in understanding the services. During the visits, HEWs/HDAs describe the colour-coded images on the tool and place the same coloured stickers on appointment days to help remind families about important health post visits for critical services. The pregnant woman, or mother, supported by the HDA, will update the calendar based on completion of each of the services.

Mama's Mataweshas complement family health cards and are used during the following periods:

1) Pregnancy: Pregnant women are visited and counselled by HEWs who use *Mama's Matawesha* to discuss antenatal care, birth planning and the importance of making sure that deliveries are managed by skilled health workers. These discussions involve other key decision-makers in the home as appropriated. HEWs explain how to use the calendar to plan for critical health services and attach stickers to indicate antenatal care appointment dates and the expected date of delivery;

2) Immediate post-delivery: Post-delivery, the HEWs immediately return to the home to discuss/plan the appointment dates for postnatal care, immunization and family planning services. HEWs discuss (and place appointment stickers for) postnatal, immunization and family planning services with new mothers and their families; specifically alerting them about date/time of the next vaccine outreach. HDAs also use the calendars during their routine home visits as a tool to discuss and track uptake of key health services and update appointment dates if necessary;

3) Immunization drop-out: In the case of missed appointments, HEWs include infants in the defaulter tracing tool and HDAs are informed to visit and counsel the caregivers on catch-up immunisation and will place an additional sticker on the calendar to indicate the date of the next vaccine outreach. HDAs also use the calendar to involve other family members in discussion about vaccination. In this way the calendar aims to address the issue that women are often not the main or only decision-makers within the household when it comes to decisions about the health of their children. That HDA will visit the same household again a few days prior to the outreach to promote attendance.

In cases where the caregivers did not receive a calendar at the time of the registration of the pregnancy HEW will conduct a home visit. They will then place appropriate stickers on a new colour coded health calendar and give the calendar to the caregiver (usually the mother) after carefully explaining the use of the calendar and the appointment dates as indicated by the stickers. Caregivers hang the calendar in their homes, so that the HEW and HDA can use them as an interpersonal communication aid during home visits. If the caregiver already has a calendar in their home, the HEW

works with the HDA to place the appropriate stickers on the immunization appointment dates during postnatal home visits. In case a mother/child pair requires two calendars (the schedule runs over the end of the 1st calendar) a 2nd calendar is given after the first one is completed.

This intervention aims to improve the quality of home visits. Home visits are intended to be interactive, motivational sessions during whtih HDAs and HEWs engage caregivers to develop a plan for preventative healthcare seeking, including catch-up vaccinations. Therefore, IRC trains HEWs directly, and facilitates HEW-led training of HDAs, on immunisation key messages, addressing myths and misconceptions, improving interpersonal communication skills for effective, tailored, counselling sessions. In addition to serving as a reminder of upcoming health appointments, the calendar tool also serves as an Information Education Communication/Behaviour Change Communication tool promoting and facilitating discussion around maternal health services, immunization and family planning during home visits conducted by the HEWs or HDAs. Refresher training sessions will be conducted at the beginning of the project.

User feedback from informal key-informant exchanges suggests that the calendars play an important role in immunisation-seeking decision-making in the household, reminding and empowering families to better plan appointments. Feedback from beneficiaries also suggests that the calendar improved the interpersonal communication between the clients and HEWs/HDAs, enabled the clients to keep track of and remember upcoming perinatal and immunization appointment dates, improved their knowledge on pregnancy danger signs and actions needed in the events of such dangers, and generally empowered mothers who use the calendars. Government health offices at the Woreda and zonal levels accepted the tool and requested its scale up. The IRC proposes to formally assess the acceptability and performance of this tool.

Appendix 3

Study Information Letter

Study Information Letter
(Version 2_. 29/02/2015)

A formative evaluation of 'The Fifth Child Project': A new initiative to close the childhood immunisation gap in Benishangul-Gumuz Regional State in Ethiopia

You are being invited to take part in an evaluation study. Before you decide it is important for you to understand why the evaluation is being done and what it will involve. Please take time to read the following information carefully and to talk to others about the study, if you wish. Ask us if there is anything that is not clear or if you would like more information. Take time to decide whether or not you wish to take part.

What is the purpose of the study?
'The Fifth Child Project' aims to increase vaccination coverage by working with local communities in a way that promotes the principle of shared responsibility for immunisation. Community leaders are encouraged to co-manage vaccination activities with health extension workers and members of the health development army, and parents and caregivers are given more opportunity to discuss, learn about and access immunisation services. New tools like a colour coded health calendar (Mama Matawesha) and a defaulter tracing tool have been developed and introduced in order to support communication and the timely uptake of vaccinations.

The purpose of this study is to evaluate the implementation and acceptability of 'The Fifth Child Project' and consider how it contributes to supporting the timely uptake of vaccines and increased coverage. The study findings will be used to improve the project and will be shared with health policy makers to inform national strategies for improving immunisation rates.

Why have I been chosen?
You are being invited to take part in this study because we would like to learn from your experience of 'The Fifth Child Project', whether as a parent/caregiver of an infant, a community leader or a health worker. We want to talk to a wide range of people including parents/caregivers of infants, health officials, immunisation coordinators, community leaders, health extension workers and members of the health development army. It is important for us to consider the perspectives and experiences of all these different people in our evaluation.

Do I have to take part?
Your participation in this study is entirely voluntary. If you are interested in taking part in the study, we will go through this information sheet with you and give you the opportunity to ask any questions you may have. If you agree to take part in an interview or focus group we will arrange to meet at a date, time and place that is convenient to you. Before the interview or focus groups starts we will ask you to confirm verbally that you are willing to take part in this study. This verbal confirmation will be recorded to ensure that you are happy to take part and know that your participation is voluntary. Even if you say yes at that point you are free to withdraw at any time, even during the interview, without giving a reason.

What will happen to me if I take part?

If you agree to take part in this study, a research assistant will arrange to meet with you at a time and place convenient for you. You will take part in an interview or a focus group with 6-10 other people, or if you are a health extension worker or a member of the health development army the research assistant may ask to accompany you on a home visit to caregivers of infants. In the interviews and focus groups, the research assistant will ask you some questions about different aspects of 'The Fifth Child Project'. For example, what you think about the colour coded health calendar and how easy it is to use, or how community leaders are involved in organising vaccine outreach activities. Your views are very important and what you tell us will help improve the project.

The interviews will take about one hour and the focus groups about an hour and a half. The research assistant will take notes and with your permission, the interview will be audiotaped. The audio-recordings from the interview will be transcribed into text, and anonymised so that the people taking part in the interview cannot be identified. We will store the interview data securely in line with Research Ethics Committee guidelines and only members of the research team will have access to this. We may use quotes from the interviews in reports and academic publications but these will be anonymous.

Expenses and payments
You should not incur any expenses from taking part in this study since the interviews will take place in your homes, at your work or at places, which are convenient for you. You will not receive any payment for taking part in this study.

What are the possible disadvantages and risks of taking part?
You may feel uncomfortable about talking about your experience but please be reassured that the aim of this study is to learn from your perspectives in order to improve the project. We will not be evaluating your work or behaviour. The research assistants, who will be interviewing you or accompanying you on home visits, do not work on the 'The Fifth Child Project' and will respect your confidentiality.

What are the possible benefits of taking part?
Taking part in the study is unlikely to benefit you directly, however the information you share with us will help inform the way future immunisation programmes are organised.

Will my taking part in the study be kept confidential?
Yes. Any information that you share with us during the course of this study will be kept strictly confidential and we will not tell anyone about your participation in this study. However, since the evaluation is being done at community level people might want to find out what you think about the study. The interviews and focus groups and accompanied visits will be conducted by research assistants, who do not work on 'The Fifth Child Project'. Interview and focus group transcripts will be made anonymous and stored securely in line with Research Ethics Committee guidelines. Where appropriate, anonymised quotes from your interview may be used in publications or reports to illustrate certain points. Upmost care will be taken to ensure that no individual or organisation can be identified in reports presented to funders and collaborators and publications disseminated at conferences, workshops and in academic journals.

What will happen to the results of the research study?
The results of this study will be written up in a report, which will be shared with people who are responsible for running and planning 'The Fifth Child Project'. This includes staff who work for IRC Ethiopia, local health officials, and researchers based at the London School of Hygiene & Tropical Medicine. The report will also be sent to the International Initiative for Impact

Evaluation (3ie) who is funding this study. We will also publish findings from our research in academic journals and comment on these on the London School of Hygiene & Tropical Medicine website. We may be asked to comment on our research and findings by representatives of the media. You will not be identified in any report, publication or media communications and we will send you a summary of our research findings and a copy of the main published paper.

Who is organising and funding the research?
This study is funded by the International Initiative for Impact Evaluation (3ie) and organised jointly by the London School of Hygiene and Tropical Medicine and the International Rescue Committee.

Who has reviewed the study?
This study was given a favourable ethical opinion by the London School of Hygiene and Tropical Medicine Research Ethics Committee, which is a committee whose task is to make sure that research participants are protected from harm. If you wish to find out more about the LSHTM Research Ethics Committee, you can contact them by email on ethics@lshtm.ac.uk. It was also approved by the Regional Health Bureau of Benishangul Gumuz Regional State.

Contact Details
If you have any practical questions about this study, please do not hesitate to contact the research assistants at the following places or numbers:

[Research Assistant Names and Contact Details to be added]

You can also contact other members of the research team in case you have any current or future concerns about the study.

Dr Tracey Chantler<br>LSHTM, 15-17 Tavistock Place<br>London, WC1H 9SH<br>England<br>Contact details were added	Emilie Karafillakis<br>LSHTM, Keppel Street<br>London, WC1E 7HT<br>England<br>Contact details were added	Shiferaw D Demissie<br>IRC Ethiopia, TK International Building, P.O. Box 107 Code 1110, Addis Adeba<br>Ethiopia<br>Contact details were added

Thank you for considering taking part in this study

If you decide to take part, please keep a copy of this information sheet for your records.

Data collection tools

Study protocol:
A formative evaluation of *'The Fifth Child Project'*: A new initiative to close the childhood immunisation gap in Benishangul-Gumuz Regional State in Ethiopia.

Version 2, dated 29/02/16. LSHTM Ethics Reference 10542, Beningshangul Gumuz Regional Health Bureau Ethics Committee Reference: 674/ቤ.ሮ m-o1

Please note these tools were translated into local languages by research assistants from the study area as part of the study training led by LSHTM investigators.

<u>Related data management</u>
The socio-demographic information collected as part of the consent process prior to the interviews, focus groups and observations was managed as described in the protocol and outline here: Each participant was allocated a numerical identifier (e.g. Community Member #1, Parent/caregiver #3, Health Care Worker #4, Community Health Worker #1, Woreda Staff #5) and only the Ethiopian investigators, the RAs and LSHTM investigators had access to the socio-demographic sheet that linked the location code and numerical identifier to a particular interviewee. Participant's socio-demographic information sheets were stored in a locked file at IRC offices, which only the RAs, the LSHTM investigators and Ethiopian investigators were able to access. Interview transcripts, field notes, scanned copies of participant's socio-demographic information and routine immunisation and project data were sent to LSHTM investigators by email as password-protected zip file attachments. These files were stored on the secure LSHTM network and only be accessed by LSHTM study investigators. The emails were destroyed as soon as the files were retrieved.

Interviews with caregivers

Research assistant interviewer(s) name(s): ____

Date: _________________ Language of interview: ____________________________

Location of interview: ____

Part A: Consent process

1. Read study information letter with participant, ensure they understand the purpose of the study, and give them the opportunity to ask questions.
2. If they are happy to take part in the study and be interviewed, ask them to state this very clearly at the beginning of the interview recording.

Confirmation of verbal consent

I explained what participation in the study will involve, gave the interviewee an opportunity to ask questions and that _______________________________ provided verbal consent to be interviewed.

Name of interviewee

_______________________________ Date: _________________

Research assistant's name

Part B: Interviewee socio-demographic characteristics

Participant number: ______ (e.g. Mugfude 1, Amba 1, Jematsa 1)

Interviewee(s) Socio-demographic characteristics

Name of Interviewee: ____

Date of interview: __________________ Place: ______________________________

Gender: Male ☐ Female ☐ Age: _____________________________

Location

Woreda: ______________________ Kebele: _____________________________

Sub-village: ____

Name of health facility in their area: ____

Relationship to infant targeted by 'Fifth Child Project Activities'

Mother ☐ Father ☐ Grandmother ☐ Grandfather ☐

Other ☐ If other, please state: ____

Details about child targeted by 'Fifth Child Project Activities':

Age (in years and months): _____________ Gender: Male ☐ Female ☐

Name of child: ____

Vaccination status (tick all that are relevant):
Up to date with scheduled immunisations ☐
Timely receipt of immunisations received ☐
Some immunisations were delayed ☐ If yes, which ones: ____________________
____

No immunisations received ☐
Missing some immunisations ☐ If yes, which ones: ____________________
____

Other children in the household that this person cares for: __________________________________
____
____
____

Educational background: ____

Able to read the Colour Coded Calendar: Yes No
Comments: ____

Where is the Colour Coded Calendar placed: ____
____

Employment/income generating activities (individual & household):
____
____
____

Relevant study objectives

1. To observe how 'The Fifth Child Project' is integrated within the local health system and document health workers/officials, community leaders and caregiver's perspectives and experiences of this project and its' contribution;
3. To observe how the 'Mama Matawesha' tool and interpersonal communication skills training are used and applied in HDA and HEW interactions with caregivers, and document HDAs and HEWs perspectives and experiences of using the tool and applying new skills;
4. To explore caregiver's perspectives and experiences of interactions with health workers and community leaders, who co-manage Fifth Child Project activities;
5. To observe how the defaulter tracing system and tool is being applied and document health workers/officials', community leaders' and caregivers' perspectives and experiences of the system and tool;

Topic Guide

Exposure to the intervention

What do you know and think about childhood immunisation? Where do you go to have your children vaccinated? *(Probes: Sources of information-health professionals, HDAs, local or other health facilities, vaccine outreaches)*

Who makes decisions about childhood immunisation in your household? *(Probes: who decides whether children are immunised, who takes them to have their vaccines?)*

What is your experience of the health development army, are you visited by a member of health development army? *(Probes: what do they tell you about childhood immunisation, what do you think about their role and input)*

What do you know about the *'Fifth Child Project' ***(*or use a term that is used locally to summarise the IRC work on immunisation)***? *(Probes: the colour coded calendar (CCC)/defaulter tracing tool (DTT), vaccine outreaches, what do you think about the project)*

Experience with the intervention

Have you or someone in your household been given a colour-coded health calendar? If yes, what do you use the calendar for and where is the calendar placed in your home? *(Probes: When were you given this, who gave it to you)*

How do you use the calendar, what does it help you with? Who else helps you to use the calendar and when do they do this and how? *(Probe: use of stickers, what aspects of the calendar do they use, the dates, the pictures)*

What do you think about the calendar, what is good, what could be improved? *(Probes: Size of calendar, size of font, acceptability of the images, health messages, use of stickers, who uses the stickers)*

What do you think about the way that health development army interact with you when they visit you in your home and talk to you about childhood immunisation? *(Probes: respect for you, addressing your concerns, supporting your decision-making, understanding your circumstances, explaining immunisation)*

Has the child in your care missed any immunisations or been delayed in receiving any immunisations? If yes, did a health worker or member of the health development army, a community leader visit you to talk to you about getting your child immunised? *(Probes: manner of interaction, how did this make you feel, what did you do as a result of this visit, were you aware that your child had missed a vaccination, who do you think should visit you-HDS, community leader, HEW, nurses)*

What do you think about the care you receive at immunisation outreach services at your health post? *(Probes: respect for you, addressing your concerns, supporting your decision-making, understanding your circumstances, explaining immunisation, waiting times in clinics)*

Have you ever taken your child to a vaccine outreach session? If yes, what do you think about these outreach sessions? *(Probes: organisation, how easily are they to access, waiting times, information provided, how you find out about them)*

Context of the intervention

What do other people in your family/village say about immunisation? How does this influence your views? *(Probes: where/when is immunisation discussed, who are the key influencers, who is listened to and why)*

What are the key barriers to the uptake of immunisation for you? *(Probes: access to health facilities, availability of vaccines, caregivers/family and work commitments)*

How has the community responded to the 'Fifth Child Project', its' approach and tools? *(Probes: acceptability of tools, comprehension of CCC)*

What do you think about community leaders' involvement in defaulter tracing, and promoting uptake of immunisation? What should their role be? *(Probes: issues of pressure and stigma)*

Any other comments?

Interviews with nurses, health extension workers & health development army leaders and members

Research assistant name(s): ____

Date of Interview: _____________ Language of interview: ______________________

Location of interview: ____

Part A: Consent process

1. Read study information letter with participant, ensure they understand the purpose of the study, and give them the opportunity to ask questions.
2. If they are happy to take part in the study and be interviewed, ask them to state this very clearly at the beginning of the interview recording.

Confirmation of verbal consent

I explained what participation in the study will involve, gave the interviewee an opportunity to ask questions and that ________________________ provided verbal consent to be interviewed.

Name of interviewee

________________________ Date: _____________

Research assistant's name

Part B: Interviewee socio-demographic characteristics

Participant number: __________________ (e.g. Mugfude 1, Amba 1, Jematsa 1)

Name of interviewee: ____

Gender: Male ☐ Female ☐ Age: ________________________

Contact details: ____

Location

Woreda: ____________________ Kebele: ________________________

Sub-village (for HDAs): ____

Health facility (for HEWS and nurses): ____

Position

Nurse: ☐ Health Extension Worker: ☐ HDAL: ☐ HDA: ☐

Length in time in current role/job: ____

Attended IRC interpersonal communication skills training (for nurses and HEWs:

Yes: ☐ No: ☐ Don't know: ☐ If yes, when and where: ______________________

Been trained by nurses or HEWs in interpersonal communication skills (for HDAs and HDALs):
Yes: ☐ No: ☐ Don't know: If yes, when and where: ______________________

Previous role/job: ____

Educational background: ____

Relevant study objectives for these interviews

1. To observe **how 'The Fifth Child Project' is integrated within the local health system and document health workers**/officials, community leaders and caregiver's **perspectives and experiences of this project and its' contribution**;
2. To explore community leaders and **health workers' views and experiences of co-managing Fifth Child Project activities**;
5. To observe how the **defaulter tracing system and tool is being applied and document health workers**/officials', community leaders' and caregivers' **perspectives and experiences of the system and tool**

Topic Guide

Implementation of the intervention

What do you know about the *'Fifth Child Project' ***(*or use a term that is used locally to summarise the IRC work on immunisation)***? *(Probes: the colour coded calendar (CCC)/defaulter tracing tool (DTT), interpersonal communication skills training)*

How is the 'Fifth Child Project' implemented in your area? *(Probes: who coordinates the project, who provides CCC, DTTs, who trains health workers on use of the tools?)*

How are local leaders (e.g. Kebele command post leaders, clan and religious leaders and school teachers) involved in the implementation of the 'Fifth Child Project' *(Probes: what do they do to support the implementation, level of interest of these leaders, tease out what community co-management means in this area)*

Tell us a bit about how and whether 'Fifth Child Project' and its' tools support immunisation activities in your area/health facility. (*Probes: contribution to achieving immunisation targets/reaching underserved groups/reducing drop-out, planning outreach events)*

Experience with the intervention

What is your role in the implementation of the 'Fifth Child Project'? *(Probes: specific tasks they do, who they report to, who they collaborate with in their locality)*

Which tools (DTT, CCC) do you use and how do you use them? What do you think about these tools?

(Probes: what works well, what is difficult, how could the tools and the way they are used be improved, how easy are they to use, work load burden)

What training have you received related to the 'Fifth Child Project'? What did you think about this training, did it help you apply the tools/communicate with caregivers and community leaders, how could the training be improved? *(Probes: Use of tools, interpersonal communication skills)*

What is your experience of using the CCC with caregivers? What is good about this tool, what could be improved, how does it help you interact with caregivers (in particular those whose infants are not up-to-date with their vaccinations)? *(Probes: access to calendars and stickers, when they are distributed, where caregivers put them in the house, who decides where the calendars are hung, who puts the stickers on the calendar, can caregivers also use the stickers)*

How is joint health worker and community leader coordination and monitoring of immunisation outreach sessions working? *(Probes: communisation, attendance at sessions)*

Tell us a bit about your opinion on how and whether the 'Fifth Child Project' tools (CCC, DTT), involvement of leaders in managing immunisation activities (e.g. joint planning of immunisation outreach events) help ensure the timely uptake of immunisation? *(Probes: what is working well, what is more challenging)*

Have you made and changes to the way that the 'Fifth Child Project' and its' constituent tools and approaches are applied in your area? (*Probes: If yes, what are these, if not what do you think could be improved and how)*

Context of the intervention

What do people in your area think about immunisation and how does this affect their decision-making about immunisation? (Probes: caregivers i.e. parents, grandparents, community leaders, who are key influencers, level of vaccine hesitancy, reasons for this hesitancy)

What are the key barriers to the uptake of immunisation in your area? *(Probes: access to health facilities, availability of vaccines, caregivers/family and work commitments)*

How have community leaders and caregivers' responded to the 'Fifth Child Project', its' approach and tools? *(Probes: acceptability of tools, comprehension of CCC)*

What do you think about community leaders' involvement in defaulter tracing, and promoting uptake of immunisation? What should their role be? *(Probes: issues of pressure and stigma, what do caregivers think about community leaders being involved)*

Have you seen an increase in understanding about immunisation and uptake of vaccines in area since 'Fifth Child Activities' started? *(Probes: what has changed-if anything, how do you know things have changed)*

Any other comments?

Interviews with community leaders and teachers

Research assistant interviewer(s) name(s): ____

Date: _________________ Language of interview: ____________________________

Location of interview: ____

Part A: Consent process

1. Read study information letter with participant, ensure they understand the purpose of the study, and give them the opportunity to ask questions.
2. If they are happy to take part in the study and be interviewed, ask them to state this very clearly at the beginning of the interview recording.

Confirmation of verbal consent

I explained what participation in the study will involve, gave the interviewee an opportunity to ask questions and that _______________________________ provided verbal consent to be interviewed.

Name of interviewee

_______________________________ Date: _________________

Research assistant's name

Part B: Interviewee socio-demographic characteristics

Participant number: ______ (e.g. Mugfude 1, Amba 1, Jematsa 1)

Interviewee(s) Socio-demographic characteristics

Name of Interviewee: ____

Date of interview: __________________ Place: ____________________________

Gender: Male ☐ Female ☐ Age: ___________________________

Location

Woreda: _______________________ Kebele: ___________________________

Command Post: ____

Name of health facility in their area: ____

Position

Kebele manager ☐ Kebele leader ☐ School Teacher (health club) ☐

Length of time performing this community role: ___________________________

Previous community role, if any: ___________________________

Other work: ___________________________

Educational background: ___________________________

Relevant study objectives

2. To observe how 'The Fifth Child Project' is integrated within the local health system and document health workers/officials, community leaders and caregiver's perspectives and experiences of this project and its' contribution;
3. To explore community leaders and health workers' views and experiences of co-managing Fifth Child Project activities;
6. To observe how the defaulter tracing system and tool is being applied and document health workers/officials', community leaders' and caregivers' perspectives and experiences of the system and tool;

Topic Guide

Implementation of the intervention

What do you know about the *'Fifth Child Project' ***(*or use a term that is used locally to summarise the IRC work on immunisation)***? *(Probes: the colour coded calendar (CCC)/defaulter tracing tool (DTT), interpersonal communication skills training, vaccine outreaches, what do you think about the project)*

How is the 'Fifth Child Project' implemented in your area? *(Probes: who coordinates the project, who carries out activities, who arranges vaccine outreaches?)*

How are you involved in the implementation of the 'Fifth Child Project'? *(Probes: what do you do to support the implementation, level of interest, can you influence how the project is implemented, have they made changes to it, try and tease out what community co-management means in this context)*

Who do you work with in the implementation of the 'Fifth Child Project' and how? *(Probes: Nurses, HEWs, HDAs, other community leaders, when to you meet, how do you collaborate, who is responsible for what, tease out how community co-management works in practice)*

Tell us a bit about what you think about how and whether 'Fifth Child Project' and its' tools support immunisation activities in your area (*Probes: contribution to achieving immunisation targets/reaching underserved groups/reducing drop-out, planning outreach events)*

Experience with the intervention

For school teachers: Tell us a little bit about how you teach students about immunisation activities? *(Probes: leaflets, understanding of colour-coded calendar)*

What do you think about the project tools (DTT, CCC), do you ever use them (if yes, how)? *(Probes: what works well, what is difficult, how could the tools and the way they are used be improved, how easy are they to use, work load burden)*

What training have you received related to the 'Fifth Child Project'? What did you think about this training, was there any opportunity to comment on the tools, vaccine outreach arrangements or suggest modifications? *(Probes: purpose and use of tools, content of interpersonal communication skills training, any input into modifying tools or supporting the IPC training)*

What is the effect of the DTT tool on planning vaccine outreach sessions? *(Probes: improved access to data, targeting sessions, collaborating with community leaders)*

How is joint health worker and community leader coordination and monitoring of immunisation outreach sessions working? *(Probes: planning, communication, increased attendance at sessions)*
For school teachers: ask about their interaction with health workers and community leaders

Tell us a bit about your opinion on how and whether the 'Fifth Child Project' tools (CCC, DTT), involvement of leaders in managing immunisation activities (e.g. joint planning of immunisation outreach events) help ensure the timely uptake of immunisation?

Have you made any changes to the way that the 'Fifth Child Project' and its' constituent tools and approaches are applied in your Kebele? (*Probes: If yes, what are these, if not what do you think could be improved and how)*

Context of the intervention

What do people in your area think about immunisation and how does this affect their decision-making about immunisation? (Probes: caregivers i.e. parents, grandparents, community leaders, who are key influencers, level of vaccine hesitancy, reasons for this hesitancy)

What are the key barriers to the uptake of immunisation in your area? *(Probes: access to health facilities, availability of vaccines, caregivers/family and work commitments)*

How have community leaders and caregivers' responded to the 'Fifth Child Project', its' approach and tools? *(Probes: acceptability of tools, comprehension of CCC)*

What do you think about community leaders'/school-teachers involvement in defaulter tracing, and promoting uptake of immunisation? What should their role be? *(Probes: issues of pressure and stigma, what do caregivers think about community leaders being involved)*

Have you seen an increase in understanding about immunisation and uptake of vaccines in area since 'Fifth Child Activities' started? *(Probes: what has changed-if anything, how do you know things have changed)*

Any other comments?

Interviews with woreda health officials

Research assistant name(s): ____

Date of Interview: _____________ Language of interview: ______________________

Location of interview: ____

Part A: Consent process

1. Read study information letter with participant, ensure they understand the purpose of the study, and give them the opportunity to ask questions.
2. If they are happy to take part in the study and be interviewed, ask them to state this very clearly at the beginning of the interview recording.

Confirmation of verbal consent

I explained what participation in the study will involve, gave the interviewee an opportunity to ask questions and that ________________________ provided verbal consent to be interviewed.

Name of interviewee

________________________ Date: _____________

Research assistant's name

Part B: Interviewee socio-demographic characteristics

Participant number: __________________ (e.g. Assosa 1, Bamabasi 1)

Name of interviewee: ____

Gender: Male ☐ Female ☐ Age: ________________________

Contact details: ____

Location of work

Woreda: ___________________________

Position

Job title: ____

Length in time in current role/job: ____

Previous role/job: ____

Educational background: ____

Relevant study objectives for these interviews

3. To observe **how 'The Fifth Child Project' is integrated within the local health system and document health workers**/officials, community leaders and caregiver's **perspectives and experiences of this project and its' contribution**;
4. To explore community leaders and **health workers' views and experiences of co-managing Fifth Child Project activities**;

6. To observe how the **defaulter tracing system and tool is being applied and document health workers**/officials', community leaders' and caregivers' **perspectives and experiences of the system and tool**

Topic Guide

Implementation of the intervention

What do you know about the *'Fifth Child Project' ***(*or use a term that is used locally to summarise the IRC work on immunisation)***? *(Probes: the colour coded calendar (CCC)/defaulter tracing tool (DTT), interpersonal communication skills training)*

How is the 'Fifth Child Project' implemented in your woreda? *(Probes: who coordinates the project, who provides CCC, DTTs, who trains health workers on use of the tools?)*

How are you, other woreda officials, health workers you supervise involved in the implementation of the 'Fifth Child Project'? *(Probes: what do they do to support the implementation, level of interest, can they influence how the project is implemented, have they made changes to it).*

How are community leaders (kebele managers/leaders, house speakers) involved in the implementation or different aspects of the 'Fifth Child Project'? *(Probes: what do they know about the project, what do they think about it, what do they do to support it, try and tease out what community co-management means in this context)*

Tell us a bit about how and whether 'Fifth Child Project' and its' tools support immunisation activities in your woreda. (*Probes: contribution to achieving immunisation targets/reaching underserved groups/reducing drop-out, planning outreach events)*

Experience with the intervention

What do you think about the project tools (DTT, CCC), do you ever use them in your work at woreda level (if yes, how)? *(Probes: what works well, what is difficult, how could the tools and the way they are used be improved, how easy are they to use, work load burden)*

What training have you received related to the 'Fifth Child Project'? What did you think about this training, was there any opportunity to comment on the tools or suggest modifications? *(Probes: purpose and use of tools, content of interpersonal communication skills training, any input into modifying tools or supporting the IPC training)*

What is the effect of the DTT tool on planning vaccine outreach sessions? *(Probes: improved access to data, targeting sessions, collaborating with community leaders)*

How is joint health worker and community leader coordination and monitoring of immunisation outreach sessions working? *(Probes: planning, communication, increased attendance at sessions)*

Tell us a bit about your opinion on how and whether the 'Fifth Child Project' tools (CCC, DTT), involvement of leaders in managing immunisation activities (e.g. joint planning of immunisation outreach events) help ensure the timely uptake of immunisation?

Have you made any changes to the way that the 'Fifth Child Project' and its' constituent tools and approaches are applied in your woreda? (*Probes: If yes, what are these, if not what do you think could be improved and how)*

Context of the intervention

What do people in your woreda think about immunisation and how does this affect their decision-making about immunisation? *(Probes: caregivers i.e. parents, grandparents, community leaders, who are key influencers, level of vaccine hesitancy, reasons for this hesitancy)*

What are the key barriers to the uptake of immunisation in your area? *(Probes: access to health facilities, availability of vaccines, caregivers/family and work commitments)*

How have community leaders and caregivers' responded to the 'Fifth Child Project', its' approach and tools? *(Probes: acceptability of tools, comprehension of CCC)*

What do you think about community leaders' involvement in defaulter tracing, and promoting uptake of immunisation? What should their role be? *(Probes: issues of pressure and stigma, what do caregivers think about community leaders being involved)*

Have you seen an increase in understanding about immunisation and uptake of vaccines in area since 'Fifth Child Activities' started? *(Probes: what has changed-if anything, how do you know things have changed?)*

What other immunisation activities are taking place in your woreda (government and NGO directed?)? *(Probes: where are these taking place, what is their aim, how do you manage these activities/ensure they complement each other)*

Any other comments?

Focus group with caregivers

Research assistant interviewer(s) name(s): ____

Date: _________________ Language of interview: ____________________________

Location of interview: ____

Part A: Consent process

1. Read study information letter with participant(s), ensure each person understands the purpose of the study, and give them the opportunity to ask questions.
2. If they are happy to take part in the study and be interviewed, ask them to state this very clearly at the beginning of the interview recording.

Confirmation of verbal consent

I explained what participation in the study will involve, gave the interviewee an opportunity to ask questions and that _______________________________ provided verbal consent to be interviewed.

Name of interviewee

_______________________________ Date: _________________

Research assistant's name

Part B: Interviewee socio-demographic characteristics

Participant number: ______ (e.g. Mugfude 1, Amba 1, Jematsa 1)

Focus group number: ______

Interviewee(s) Socio-demographic characteristics

Name of Interviewee: ____

Date of interview: __________________ Place: ______________________________

Gender: Male ☐ Female ☐ Age: _____________________________

Location

Woreda: ______________________ Kebele: _____________________________

Sub-village: ____

Name of health facility in their area: ____

Relationship to infant targeted by 'Fifth Child Project Activities'

Mother ☐ Father ☐ Grandmother ☐ Grandfather ☐

Other ☐ If other, please state: ____

Details about child targeted by 'Fifth Child Project Activities':

Age (in years and months): ____________ Gender: Male ☐ Female ☐

Name of child: ____

Vaccination status (tick all that are relevant):

Up to date with scheduled immunisations ☐

Timely receipt of immunisations received ☐

Some immunisations were delayed ☐ If yes, which ones: ____________________

____

No immunisations received ☐

Missing some immunisations ☐ If yes, which ones: ____________________

____

Other children in the household that this person cares for: _________________________________

____

____

____

Educational background: ____

Able to read the Colour Coded Calendar: Yes No

Comments: ____

Employment/income generating activities (individual & household):

____

____

____

Relevant study objectives

4. To observe how 'The Fifth Child Project' is integrated within the local health system and document health workers/officials, community leaders and caregiver's perspectives and experiences of this project and its' contribution;
5. To observe how the 'Mama Matawesha' tool and interpersonal communication skills training are used and applied in HDA and HEW interactions with caregivers, and document HDAs and HEWs perspectives and experiences of using the tool and applying new skills;
6. To explore caregiver's perspectives and experiences of interactions with health workers and community leaders, who co-manage Fifth Child Project activities;
7. To observe how the defaulter tracing system and tool is being applied and document health workers/officials', community leaders' and caregivers' perspectives and experiences of the system and tool;

Topic Guide

Exposure to the intervention

What health activities take place at your local health post? What do you think about the immunisation activities that take place at your health post, or in your area? (*Probes: availability of vaccines, skill of HEWs, information and communication about vaccines, quality of service)*

What do you know and think about childhood immunisation? Where do you go to have your children vaccinated? *(Probes: Sources of information-health professionals, HDAs, local or other health facilities, vaccine outreaches)*

Who makes decisions about childhood immunisation in your households? *(Probes: who decides whether children are immunised, who takes them to have their vaccines?)*

What is your experience of the health development army, are you visited by a member of health development army? *(Probes: what do they tell you about childhood immunisation, what do you think about their role and input)*

What do you know about the colour coded-calendar and other community based immunisation activities? *(Probes: vaccine outreaches, leaders encouraging you to vaccinate your children)*

Experience with the intervention

Show the colour-coded calendar

Have you ever seen this calendar? Have you been given one, if yes by whom, when and why? What do you think about the calendar, what is its purpose, what is good, what could be improved? *(Probes: What do you think about the presentation of dates, what do you think about the pictures, size of calendar, size of font, acceptability of the images, health messages)*

How do you or others use the calendar, how does it help you or them? Who else helps you to use the calendar and when do they do this and how? *(Probe: use of stickers, what aspects of the calendar do they use, the dates, the pictures)*

What do you think about the way that health development army interact with you when they visit you in your home and talk to you about childhood immunisation? *(Probes: respect for you, addressing your concerns, supporting your decision-making, understanding your circumstances, explaining immunisation)*

What happens if children have missed or been delayed in receiving any immunisations? *(Probes: Are you visited in your homes, by whom, what are these visits like, manner of interaction between health workers and caregivers?)*

Have you ever taken your child to a vaccine outreach session, what do you think about the care you receive at your health post, during vaccine outreaches and at other times? *(Probes: how easy they are to access, respect for you, addressing your concerns, supporting your decision-making, understanding your circumstances, explaining immunisation, waiting times in clinics)*

What do you think about community leaders' involvement in defaulter tracing, and promoting uptake of immunisation? What should their role be? *(Probes: issues of pressure and stigma)*

Context of the intervention

What do other people in your family/village say about immunisation? How does this influence your views? *(Probes: where/when is immunisation discussed, who are the key influencers, who is listened to and why)*

What are some of the key barriers to the uptake of immunisation? *(Probes: access to health facilities, availability of vaccines, caregivers/family and work commitments)*

How has the community responded to the colour-coded calendar and defaulter tracing? *(Probes: acceptability of tools, comprehension of CCC)*

Any other comments?

Focus group guide for health development army members

Research assistant name(s): ____

Date of Interview: _______________ Language of interview: ____________________

Location of interview: ____

Part A: Consent process

1. Read study information letter with participant (s), ensure each person understands the purpose of the study, and give them the opportunity to ask questions.
2. If they are happy to take part in the study and be interviewed, ask them to state this very clearly at the beginning of the interview recording.

Confirmation of verbal consent

I explained what participation in the study will involve, gave the interviewee an opportunity to ask questions and that ____________________ provided verbal consent to be interviewed.

Name of interviewee

____________________ Date: _______________

Research assistant's name

Part B: Interviewee socio-demographic characteristics

Participant number: _______________ (e.g. Mugfude 1, Amba 1, Jematsa 1)

Focus group number: _______________

Name of interviewee: ____

Gender: Male ☐ Female ☐ Age: ____________________

Contact details: ____

Location

Woreda: ____________________ Kebele: ____________________

Sub-village: ____

Position

HDAL: ☐ HDA: ☐

Length in time in current role/job: ____

Received training by nurses or HEWs in interpersonal communication skills:

Yes: ☐ No: ☐ Don't know: If yes, when and where: ______________________

Previous role/job: ____

Educational background: ____

Relevant study objectives for these interviews

5. To observe **how 'The Fifth Child Project' is integrated within the local health system and document health workers**/officials, community leaders and caregiver's **perspectives and experiences of this project and its' contribution**;
6. To explore community leaders and **health workers' views and experiences of co-managing Fifth Child Project activities**;
7. To observe how the **defaulter tracing system and tool is being applied and document health workers**/officials', community leaders' and caregivers' **perspectives and experiences of the system and tool**

Topic Guide

Implementation of the intervention(s)

What health activities take place at your local health post? What do you think about the immunisation activities that take place at your health post, or in your area? (*Probes: availability of vaccines, skill of HEWs, information and communication about vaccines, quality of service)*

How are immunisation activities implemented in your area? *(Probes: who coordinates the activities, who provides you with CCC, DTTs, who trains you on use of the tools?)*

What do you know and think about the colour-coded calendar? What do you know and think about the DTT? *(Probes: Have you used these tools, what did they help you do?)*

How are local leaders (e.g. Kebele command post leaders and schoolteachers) involved in the implementation of immunisation activities? *(Probes: what do they do to support the implementation, level of interest of these leaders, tease out what community co-management means in this area)*

Experience with the intervention

What immunisation activities are you involved in? *(Probes: specific tasks they do e.g. distribute CCC use DTT to find defaulters, counselling, who they report to, who they collaborate with in their locality)*

Use of (CCC & DTT): how do you use these tools? What do you think about these tools? *(Probes: what works well, what is difficult, how could the tools and the way they are used be improved, how easy are they to use, work load burden)*

Encourage participants to tell you about real life experiences of using the calendars and DTT. (Try and get them to talk about a range of cases-when the calendar was first introduced, with people who did not understand it, with people who were positive about it)

Show participants a calendar and ask them to comment on what is good about the calendar, and what could be improved. (Do the same for the DTT)

What training have you received related to the 'Fifth Child Project'? What did you think about this training, did it help you apply the tools/communicate with caregivers and community leaders, how could the training be improved? *(Probes: Use of tools, interpersonal communication skills)*

What is your experience of using the CCC with caregivers? What is good about this tool, what could be improved, how does it help you interact with caregivers (in particular those whose infants are not up-to-date with their vaccinations)? *(Probes: access to calendars and stickers, when they are distributed, where caregivers put them in the house, who decides where the calendars are hung, who puts the stickers on the calendar, can caregivers also use the stickers)*

Tell us about how and whether the tools (CCC, DTT) and involvement of leaders in managing immunisation activities (e.g. joint planning of immunisation outreach events), help ensure the timely uptake of immunisation? *(Probes: what is working well, what is more challenging, how do tools help meet immunisation targets)*

Have you made any changes to the way that you use the calendar and DTT and work with local leaders in your area? (*Probes: If yes, what are these, if not what do you think could be improved)*

What is your experience of supervision, who supervises you, how do they supervise you, what is this like for you? *(Probes: Frequency of supervision, quality of supervision, how does supervision help you improve your practice?)*

How do you work with other members of the HDA, other health workers and community leaders? *(Probe: what is the nature of this collaboration, how could it be improved, what is good about this teamwork?)*

Context of the intervention

What do people in your area think about immunisation and how does this affect their decision-making about immunisation? (*Probes: caregivers i.e. parents, grandparents, community leaders, who are key influencers, level of vaccine hesitancy, reasons for this hesitancy)*

What are the key barriers to the uptake of immunisation in your area? *(Probes: access to health facilities, availability of vaccines, caregivers/family and work commitments)*

How have community leaders and caregivers' responded to the colour-coded calendar, the DTT, and the immunisation counselling? *(Probes: acceptability of tools, comprehension of CCC, interaction with HDAs and HEWs)*

What do you think about community leaders' involvement in defaulter tracing, and promoting uptake of immunisation? What should their role be? *(Probes: issues of pressure and stigma, what do caregivers think about community leaders being involved)*

Have you seen an increase in understanding about immunisation and uptake of vaccines in your area since you started to use the calendars and DTT? *(Probes: what has changed-if anything, how do you know things have changed?)*

Any other comments?

Field notes observation template

Research assistant's name: ____

Approval obtained from relevant people to observe the event.

______________________________ ________________

Research assistant's signature Date

Event observed: ____

Date and time of event: ____

Place of event: ____

Description of the context:

____

____

____

____

____

____

____

Description of people present (also estimate numbers of different people present):

____

____

____

____

____

____

____

Acts *(what is happening, what is the purpose of the event?)*:

____

____

____

____

____

Activities *(what are people doing, what is covered in the meeting?)*:

Participation (who is participating, who is making decisions about what is happening, how are health workers and VHTs involved, how are community leaders involved?):

Relationships (how are people interacting, what are they saying):

Who did you interact with during this event, why and what did you talk about?

What did you learn from this event (reflections on what happened in terms of the aims of the project)?

Home visit observation field notes sheet and checklist

Research assistant's name: ____

Consent obtained from the HEW/HDA to join them on the visit, and the HEW/HDA has informed the caregiver that you will be joining them on the home visit and they have given their consent for you to visit their home.

_____________________________ _____________

Research assistant's signature Date

Name of HEW/HDA they are accompanying: ____________________________________

Kebele: ____

Caregivers name where home visit is taking place: ______________________________

Date and time of visit: ____

Relationship to infant targeted by 'Fifth Child Project Activities'

Mother ☐ Father ☐ Grandmother ☐ Grandfather ☐

Other ☐ If other, please state: ______________________________________

Details about child targeted by 'Fifth Child Project Activities':

Age (in years and months): ___________ Gender: Male ☐ Female ☐

Name of child: ____

Vaccination status (tick all that are relevant):

Up to date with scheduled immunisations ☐

Timely receipt of immunisations received ☐

Some immunisations were delayed ☐ If yes, which ones: _____________

____

No immunisations received ☐

Missing some immunisations ☐ If yes, which ones: _____________

____

Other children in the household that this person cares for: _____________________

____

Educational background: ____

Able to read the Colour Coded Calendar: Yes No

Comments: ____

State the purpose of the visit as determined by the HEW or HDA:

____
____
____
____
____

Describe the context of the visit (Size of house and compound, activities the household is involved in e.g. farming, other):

____
____
____
____

State who is present during the home visit and how involved they are in the visit:

____
____
____
____
____

State where the calendar is placed in the home (e.g. hung on the wall, door, or kept folded up):

____
____
____
____

Visit checklist adapted from the training manual

**Checklist Prior to Visit**	**Yes**	**No**	**Comment**
Review key messages to be delivered, and action that they are trying to encourage			
Are any additional materials needed?			
**Checklist during the Visit**	**Yes**	**No**	**Comment**
**Creating a Caring Environment Skill: Building rapport**			
Greeting (verbal/non-verbal)			
Keeps parents relaxed –use of body language: smile, eye contact			
Soft tone, explains visits, shows empathy			

**Gathering information Skill: Questions and listens**			
Uses the appropriate questions and listens			
Encourages dialogue			
Makes eye contact, acknowledges			
Seeks more information (probes)			
Reflects feelings			
**Counseling Effectively Skill: Counseling and sharing information**			
Asks client's understanding of immunization and FP			
Discusses and tries to correct any misconceptions or rumors			
Uses simple and understandable language			
Asks for any questions or concerns			
Uses visual aids where needed			
Provides guidance on positive health action clients could take (e.g. attend vaccine outreach)			
Asks client to repeat what they need to do			
Summarizes and repeats key information			
**Checklist after the Visit**	**Yes**	**No**	**Comment**
Did the health worker reflect on whether the key messages delivered, and whether the client understand the health action they were being encouraged to take? How are they going to check if the health actions is taken?			
Did the health worker consider how the visit could have been improved, if other strategies might have been useful?			

Observer's reflections of the visit:

What went well, how engaged the caregiver (or others) was in the discussion, how the calendar or DTT was used, what did not go so well and why, what could have been done to improve the visit.

Observers notes on any interactions they have with caregivers after the visit

Use and understanding of the calendar, views on immunization and immunization services, access to health post, interactions with HEWs and HDAs
