# Supplementary figures and images for: ‘We All Work Together to Vaccinate the Child’: A Formative Evaluation of a Community-Engagement Strategy Aimed at Closing the Immunization Gap in North-West Ethiopia
Source: Int J Environ Res Public Health. 2018 Apr 3;15(4):667. doi: 10.3390/ijerph15040667 (PMC5923709; doi:10.3390/ijerph15040667)

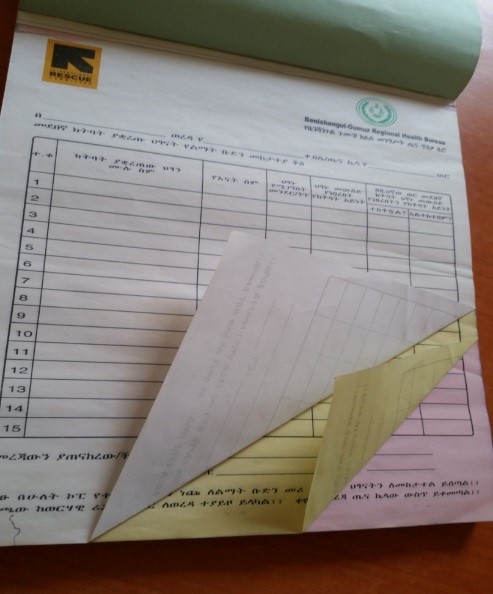

Supplement: Supplementary file 1 [file ijerph-15-00667-s001.zip › Supplementary material_Defaulter tracing tool.jpg]

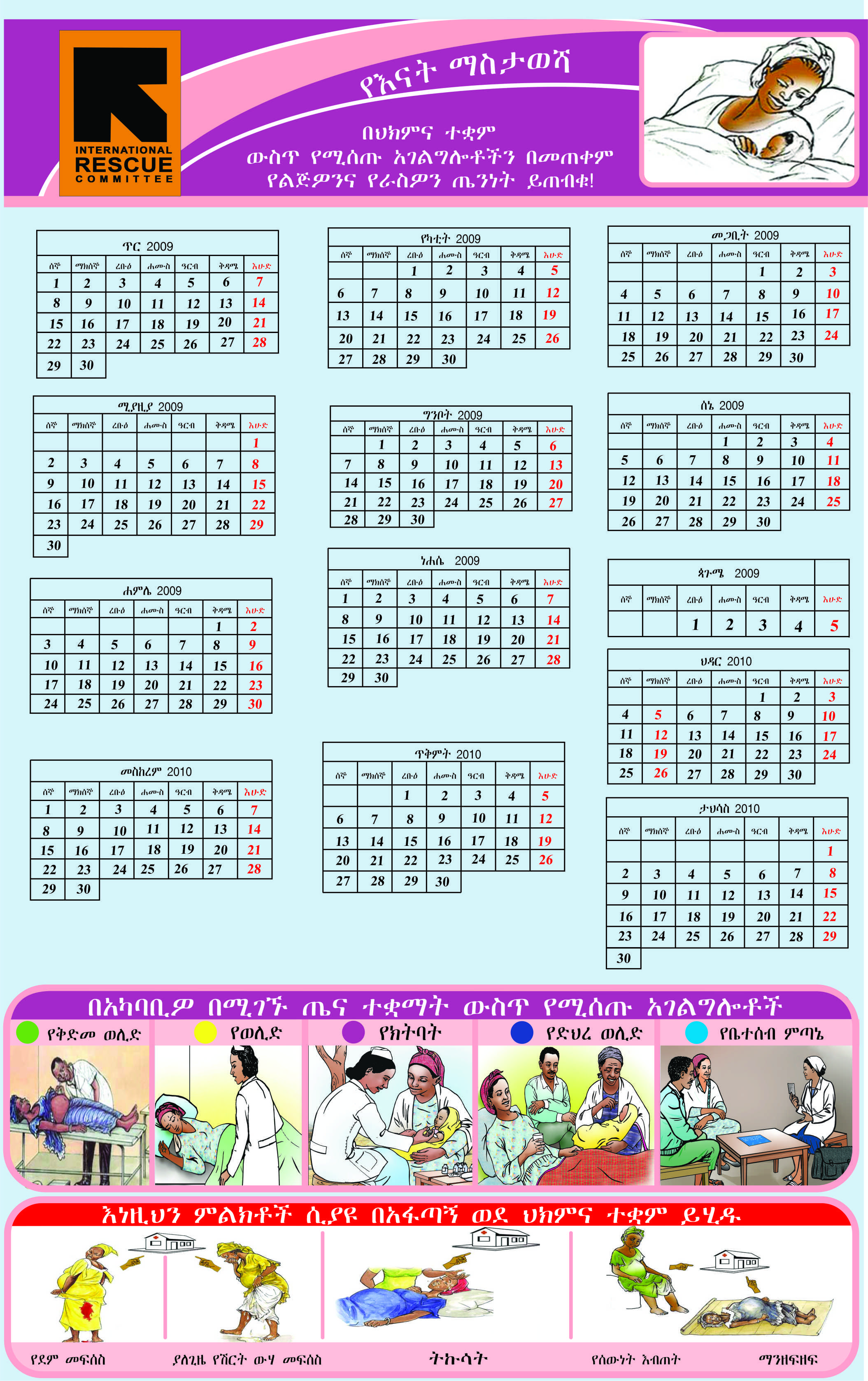

Supplement: Supplementary file 1 [file ijerph-15-00667-s001.zip › Supplementary material_Enat mastawesha calendar.jpg]
